# Genome-wide investigation reveals high evolutionary rates in annual model plants

**DOI:** 10.1186/1471-2229-10-242

**Published:** 2010-11-09

**Authors:** Jia-Xing Yue, Jinpeng Li, Dan Wang, Hitoshi Araki, Dacheng Tian, Sihai Yang

**Affiliations:** 1 State Key Laboratory of Pharmaceutical Biotechnology, School of Life Sciences, Nanjing University, 210093, Nanjing, PR China; 2 Eawag, Swiss Federal Institute of Aquatic Science and Technology, Center of Ecology, Evolution and Biogeochemistry, 6047 Kastanienbaum, Switzerland; 3 Department of Ecology and Evolutionary Biology, Rice University, Houston, TX 77005, USA

## Abstract

**Background:**

Rates of molecular evolution vary widely among species. While significant deviations from molecular clock have been found in many taxa, effects of life histories on molecular evolution are not fully understood. In plants, annual/perennial life history traits have long been suspected to influence the evolutionary rates at the molecular level. To date, however, the number of genes investigated on this subject is limited and the conclusions are mixed. To evaluate the possible heterogeneity in evolutionary rates between annual and perennial plants at the genomic level, we investigated 85 nuclear housekeeping genes, 10 non-housekeeping families, and 34 chloroplast genes using the genomic data from model plants including *Arabidopsis thaliana *and *Medicago truncatula *for annuals and grape (*Vitis vinifera*) and popular (*Populus trichocarpa*) for perennials.

**Results:**

According to the cross-comparisons among the four species, 74-82% of the nuclear genes and 71-97% of the chloroplast genes suggested higher rates of molecular evolution in the two annuals than those in the two perennials. The significant heterogeneity in evolutionary rate between annuals and perennials was consistently found both in nonsynonymous sites and synonymous sites. While a linear correlation of evolutionary rates in orthologous genes between species was observed in nonsynonymous sites, the correlation was weak or invisible in synonymous sites. This tendency was clearer in nuclear genes than in chloroplast genes, in which the overall evolutionary rate was small. The slope of the regression line was consistently lower than unity, further confirming the higher evolutionary rate in annuals at the genomic level.

**Conclusions:**

The higher evolutionary rate in annuals than in perennials appears to be a universal phenomenon both in nuclear and chloroplast genomes in the four dicot model plants we investigated. Therefore, such heterogeneity in evolutionary rate should result from factors that have genome-wide influence, most likely those associated with annual/perennial life history. Although we acknowledge current limitations of this kind of study, mainly due to a small sample size available and a distant taxonomic relationship of the model organisms, our results indicate that the genome-wide survey is a promising approach toward further understanding of the mechanism determining the molecular evolutionary rate at the genomic level.

## Background

Clarification of the pattern and dynamics of nucleotide change in evolution is of fundamental importance for understanding evolutionary mechanisms. One major focus is the determination and evaluation of factors that affect the evolutionary rate at the molecular level. While molecular clock has been largely accepted as a null hypothesis of molecular evolution, many possible influencing factors have been proposed and investigated either in animals or in plants during these decades, including changes in population size, protein dispensability, efficiency of DNA replication and repair systems, metabolic rate, speciation rate, and generation time [1-9].

Among these, life history traits are of particular interest. For example, generation time, referring to the time to reach sexual maturity, has long been suspected as a major factor that alters the molecular evolutionary rate among species. In animals, several empirical studies suggested that rodents have a higher evolutionary tempo than primates, indicating an inverse relationship between evolutionary rate and generation time [5,6,10]. In plants, on the other hand, the role of life history in molecular evolution is still under debate despite a number of studies supporting its importance [7,11-13]. Whittle and Johnston [14] reported genetic comparisons of the nuclear 18S ITS1 and ITS2 regions between annual plants and perennial plants and found no evidence for the inverse relationship between evolutionary rate and life span. Such a study raised a question against the general applicability of the generation time effect on molecular evolution of the plants. Moreover, considerable variations in breeding system, population size, speciation rate and gene-specific selective constraints introduce further complications in addressing the connection between plants' evolutionary rate and their annual/perennial habits.

Notably, one critical problem in previous studies is the limited DNA sequence data from different species and particularly different gene loci sampled, due to historical and technological reasons. Fortunately, the recent development of the DNA sequencing technology lends the resources needed to further investigate this perplexing topic based on far more sequence data than ever before. For instance, an exemplary study carried by Smith and Donoghue recently detected the much higher rates of molecular change in annuals by sampling and sequencing multiple loci from many species representing the five major branches within flowering plants [15].

Herein, we present our study on this topic from a comparative genomic perspective. Recently the whole genome sequences of many plants, ranging from moss to flowering plants including annuals and perennials, became available [16-19]. Such genomic information from multiple species offers a great opportunity to explore the heterogeneity in evolutionary rate between annuals and perennials across a large set of gene loci at the whole genome level. In this study, we investigate a total of 441 kb-long genomic sequence including 85 nuclear housekeeping genes, 10 non-housekeeping gene families and 34 chloroplast genes in four model species of dicot plants (two annuals and two perennials, Additional file 1). They include *Arabidopsis thaliana *(*At*, annual), *Medicago truncatula *(*Mt*, annual), *Vitis vinifera *(*Vv*, perennial), and *Populus trichocarpa *(*Pt*, perennial). A monocot species, *Oryza sativa *(*Os*), was used as an outgroup. A relative measure of the evolutionary rates of the orthologous genes between annuals and perennials enabled us to effectively disentangle the genome-wide effect of plant life history trait on the evolutionary rate from locus-specific effects of evolutionary forces such as protein dispensability or selective constraints. Our results provide a strong support for the association between the annual/perennial life histories of plants and the rate of molecular evolution at the genomic level.

## Results

### Phylogenetic Reconstruction

**Figure 1:**
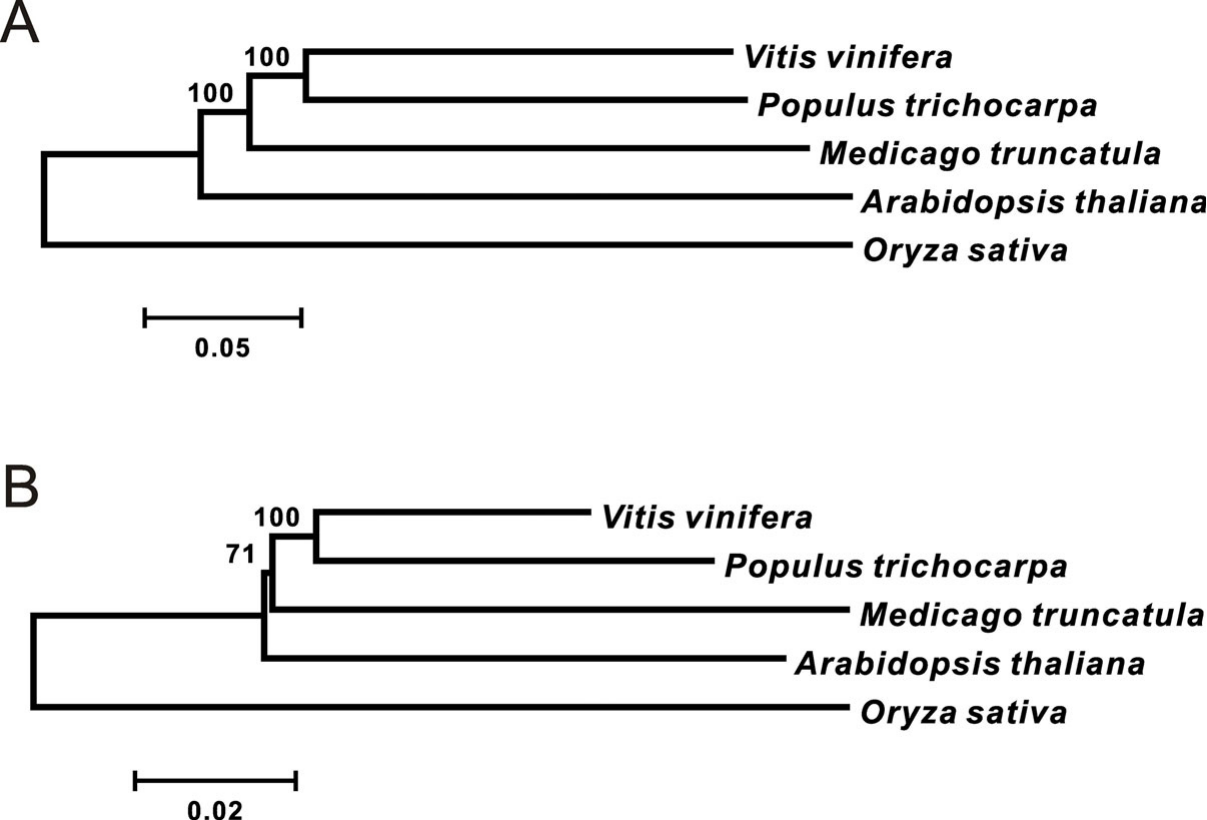
**Phylogenetic relationship of 5 plant species used in our study**. Evolutionary history of five studied species was inferred based on the nucleotide sequences of 85 nuclear genes (A) and 34 chloroplast genes (B). Neighbor-Joining method was used to reconstruct the phylogenetically consensus trees. The percentage of the bootstrap supporting the cluster is shown next to the branches (out of 1000 replicates). The trees were drawn using MEGA v4.0.

For phylogenetic reconstruction, 34 chloroplast genes were concatenated to form a 31,752 bp-long sequence data set. For nuclear genes, a data set containing 85 housekeeping genes with a total length of 79,866 bp was used (Additional file 1 for the list of genes). Well-consistent branching order was observed in the phylogenetic trees based on both datasets, although the bootstrap support of the topology was only strong for nuclear genes (Figure 1, see Methods for further details). According to the phylogenetic trees, *A. thaliana *diverged first after the split of monocots and dicots, and the two perennial species, grape (*V. vinifera*) and poplar (*P. trichocarpa*), diverged most recently. Long external branches for all five species imply that they are distantly related. In addition, the branch lengths were longer for *A. thaliana and M. truncatula *compared with *V. vinifera *and *P. trichocarpa *in both phylogenetic trees. Thus, higher evolutionary rate in annuals than in perennials, in both nuclear genes and chloroplast genes, was supported in the studied species. Detailed results for each type of genes are described in the following sections.

### Nuclear Housekeeping Genes

Using the maximum likelihood (ML) method, we found that 76.5-82.4% of the 85 nuclear housekeeping genes had higher nucleotide substitution rates (d) in annuals than in perennials in our annual-perennial pairwise comparisons (Table 1). This pattern was consistent for nonsynonymous substitution rates (d_N_) and synonymous substitution rates (d_S_) as well, with the higher-rate proportions of 62.4-74.1% (in d_N_) and 74.1-82.4% (in d_S_). The predominant heterogeneity in evolutionary rate between annual and perennial plants was statistically supported by sign-test (Table 1). Paired *t*-test was then performed to take into account the degree of annual-perennial evolutionary rate shift across each locus and allow quantitative statistic analysis. Again, the 12 cross-comparisons based on d, d_N _and d_S _showed significant annual-perennial heterogeneity in evolutionary rates (Table 2).

**Table 1:** Sign-test for the annual-perennial comparison of evolutionary rates, estimated by the ML method

Nuclear Housekeeping Genes (85 Loci)						
**Comparison Pair**	**d**		**d**_**N**_		**d**_**S**_	
			
	**Proportion**	**P-Value**	**Proportion**	**P-Value**	**Proportion**	**P-Value**

***At *vs. *Vv***	82.4%	5.86E-10	74.1%	4.91E-06	78.8%	4.21E-08
***At *vs. *Pt***	81.2%	2.62E-09	68.2%	5.08E-03	82.4%	5.86E-10
***Mt *vs. *Vv***	81.2%	2.62E-09	74.1%	4.91E-06	78.8%	4.21E-08
***Mt *vs. *Pt***	76.5%	5.15E-07	62.4%	0.015	74.1%	4.91E-06

**Non-Housekeeping Gene Families (111 Clades)**						

**Comparison Pair**	**d**		**d**_**N**_		**d**_**S**_	
			
	**Proportion**	**P-Value**	**Proportion**	**P-Value**	**Proportion**	**P-Value**

***At *vs. *Vv***	77.5%	2.52E-09	59.5%	0.029	78.4%	7.09E-10
***At *vs. *Pt***	73.9%	2.45E-07	56.8%	0.092	79.3%	1.90E-10
***Mt *vs. *Vv***	80.2%	4.82E-11	80.2%	4.82E-11	76.6%	8.49E-09
***Mt *vs. *Pt***	77.5%	2.52E-09	71.2%	4.73E-06	74.8%	8.38E-08

**Chloroplast Genes (34 Loci)**						

**Comparison Pair**	**d**		**d**_**N**_		**d**_**S**_	
			
	**Proportion**	**P-Value**	**Proportion**	**P-Value**	**Proportion**	**P-Value**

***At vs. Vv***	88.2%	3.08E-06	73.5%	4.52E-03	91.2%	3.83E-07
***At vs. Pt***	71.4%	0.012	64.7%	0.061	76.5%	1.47E-03
***Mt vs. Vv***	97.1%	2.04E-09	91.2%	3.83E-07	97.1%	2.04E-09
***Mt vs. Pt***	97.1%	2.04E-09	85.3%	1.93E-05	94.1%	3.47E-08

The proportion of genes showing higher evolutionary rate in annuals than in perennials and the *P*-value of sign-test in three different measures of the evolutionary rate (d, d_N _and d_S_) are listed in the table.

**Table 2:** *P*-values by the paired *t*-test for the heterogeneity of evolutionary rates

Nuclear Housekeeping Genes (85 Loci)						
**Comparison Pair**	**d**		**d**_**N**_		**d**_**S**_	
			
	***Vv***	***Pt***	***Vv***	***Pt***	***Vv***	***Pt***

***At***	5.09E-05	6.72E-04	1.66E-06	0.011	3.29E-06	1.37E-04
***Mt***	1.16E-06	7.74E-04	3.60E-08	3.07E-03	2.26E-05	1.59E-03

**Non-Housekeeping Gene Families (111 Clades)**						

**Comparison Pair**	**d**		**d**_**N**_		**d**_**S**_	
			
	***Vv***	***Pt***	***Vv***	***Pt***	***Vv***	***Pt***

***At***	6.12E-07	6.24E-05	1.22E-03	0.045	6.58E-06	6.66E-05
***Mt***	5.32E-09	7.45E-06	1.07E-07	1.55E-05	2.79E-07	1.12E-03

**Chloroplast Genes (34 Loci)**						

**Comparison Pair**	**d**		**d**_**N**_		**d**_**S**_	
			
	***Vv***	***Pt***	***Vv***	***Pt***	***Vv***	***Pt***

***At***	8.49E-08	8.09E-08	5.77E-03	0.243	1.48E-07	4.79E-04
***Mt***	1.22E-12	3.98E-10	2.00E-04	4.66E-03	2.96E-12	2.12E-09

The *P*-values of paired *t*-test in all 4 annual-perennial cross-comparisons suggest higher evolutionary rates in annuals than in perennials. The estimation of evolutionary rate is based on the ML method.

**Figure 2:**
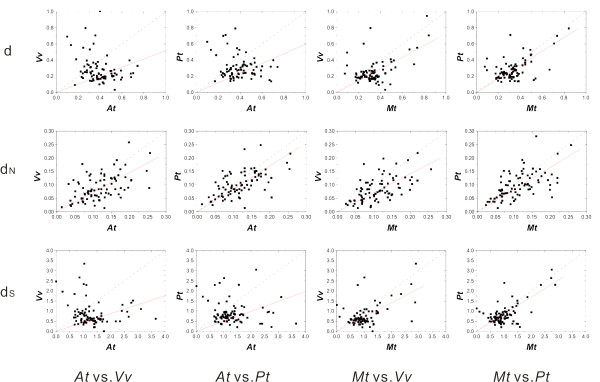
**Scatter plots of evolutionary rate in annuals against that in perennials for nuclear housekeeping genes estimated by the ML method**. Cases in all 4 annual-perennial cross-comparison are shown. The dotted line is the diagonal line with a slope equal to 1, and the red line is the regression line.

Next, we plotted evolutionary rate in annuals against that in perennials (Figure 2). We also conducted regression analysis for d, d_N _and d_S_. Majority of the plots were located below the diagonal line and the regression slope was consistently lower than unity (0.44-0.86), confirming the higher evolutionary rate in annuals than in perennials across multiple gene loci. Another interesting finding lies in the correlation of evolutionary rates between annuals and perennials, which can be considered as a measure of relative rate consistency across multiple gene loci (Table 3). For *M. truncatula*, clear correlation was detected consistently among d, d_N _and also d_S_. For *A. thaliana*, on the other hand, such correlation could be only found in d_N_, probably due to the saturation effect of synonymous substitution at some gene loci. Saturation of nucleotide substitutions can bias the estimate of evolutionary rate when evolutionary rate is high and the lineages of comparison are distantly related. Thus, the saturation effect explains the observation well, considering the early split of *A. thaliana *(Figure 1) and high nucleotide substitution rate at the synonymous sites (as indicated by d_S _> 1 in Figure 2). Indeed, out of the 85 gene loci, 34 (40%) showed d_S _>1 in the *M. truncatula *branch relative to those two perennials, whereas 63 genes (74%) showed d_S _>1 in *A. thaliana*. Although all the results above are based on the estimation of evolutionary rates using ML method, another (outgroup-dependant) method based on an assumption of molecular clock also provided similar results (See Methods, Table S1-S3 in Additional file 1, Figure S1 and S2 in Additional file 2).

**Table 3:** Correlations between annual-perennial evolutionary rates

Nuclear Housekeeping Genes (85 Loci)						
**Comparison Pair**	**d**		**d**_**N**_		**d**_**S**_	
			
	**R**^**2**^	**Slope**	**R**^**2**^	**Slope**	**R**^**2**^	**Slope**

***At vs. Vv***	0.023	0.518	0.280	0.728	0.007	0.441
***At vs. Pt***	0.008	0.597	0.375	0.855	0.005	0.493
***Mt vs. Vv***	0.251	0.725	0.350	0.707	0.261	0.703
***Mt vs. Pt***	0.404	0.852	0.365	0.825	0.501	0.849

**Non-Housekeeping Gene Families (111 Clades)**						

**Comparison Pair**	**d**		**d**_**N**_		**d**_**S**_	
			
	**R**^**2**^	**Slope**	**R**^**2**^	**Slope**	**R**^**2**^	**Slope**

***At vs. Vv***	0.075	0.588	0.548	0.842	0.008	0.505
***At vs. Pt***	0.108	0.657	0.593	0.892	0.051	0.572
***Mt vs. Vv***	0.458	0.732	0.442	0.697	0.366	0.738
***Mt vs. Pt***	0.572	0.838	0.442	0.743	0.449	0.859

**Chloroplast Genes (34 Loci)**						

**Comparison Pair**	**d**		**d**_**N**_		**d**_**S**_	
			
	**R**^**2**^	**Slope**	**R**^**2**^	**Slope**	**R**^**2**^	**Slope**

***At vs. Vv***	0.482	0.546	0.593	0.668	0.254	0.474
***At vs. Pt***	0.497	0.730	0.721	0.862	0.196	0.632
***Mt vs. Vv***	0.358	0.451	0.297	0.541	0.143	0.427
***Mt vs. Pt***	0.531	0.627	0.398	0.681	0.292	0.604

The square of correlation coefficient (R^2^) and the slope of regression line of evolutionary rate in annuals against that in perennials are listed in the table.

### Non-housekeeping gene families

Housekeeping genes are often highly conserved. Many non-housekeeping genes, on the other hand, often vary in size and functional constraint across different species. They also frequently experience duplication, recombination and diversifying selection, making their evolutionary histories much more complex than those of housekeeping genes [20]. So, it is tempting to ask whether such annual-perennial rate heterogeneity holds the same for non-housekeeping gene families. To examine this, we expanded our analysis to incorporate 10 non-housekeeping gene families with different sizes and diverse functions. A total of 111 orthologous gene clades were sampled from the 10 gene families according to our sampled criteria. Eventually four data sets were built for further analysis on the gene families (one total dataset and three sub-datasets, see Methods for detail).

**Figure 3:**
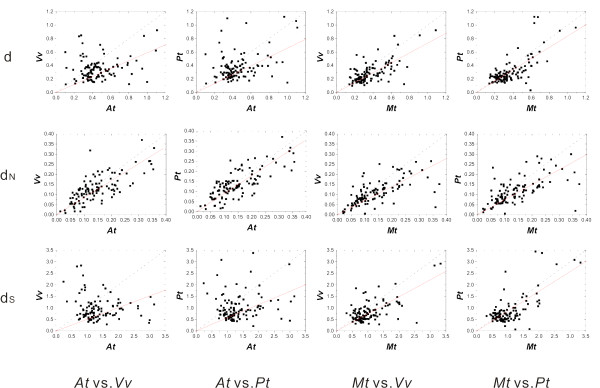
**Scatter plots of evolutionary rate in annuals against that in perennials for non-housekeeping gene families estimated by the ML method**. Cases in all 4 annual-perennial cross-comparison are shown. The dotted line is the diagonal line with a slope equal to 1, and the red line is the regression line.

The results from this analysis were largely consistent with those from housekeeping genes above, in support of heterogeneity in annual-perennial evolutionary rates even in these gene families. Both sign-test and paired *t*-test suggested significantly higher evolutionary rates in these 10 gene families (Table 1, 2 and Additional file 1). In the scatter plot for the evolutionary rates of annuals vs. perennials, regression lines clearly deviated from the diagonal line and inclined to the annual side (Figure 3 and Table 3, see also Additional files 1 and 2). Moreover, as we found in housekeeping genes, strong correlation between evolutionary rates in the annual-perennial comparisons was detected in d_N_, whereas d and d_S _showed weak or invisible correlation between *A. thaliana *and those two perennials in many cases (Table S9 in Additional file 1).

### Chloroplast genes

Previous studies indicated that nucleotide substitution rates vary greatly among nuclear, mitochondrial, and chloroplast genomes and that chloroplast genome evolve much more slowly than nuclear genome in plants [7,21]. Consistent with these reports, our analysis based on 34 randomly sampled chloroplast genes showed a considerably lower evolutionary rate than that detected in nuclear housekeeping genes and gene families (about 1/3 at nonsynonymous sites and 1/4 at synonymous sites). Therefore, for each annual-perennial comparison, the genetic divergence in chloroplast genomes tends to be much smaller than in nuclear genomes (Figure 1). Thus, we expected that chloroplast genes provide a better resolution in order to better estimate the evolutionary rates in these distantly related species.

**Figure 4:**
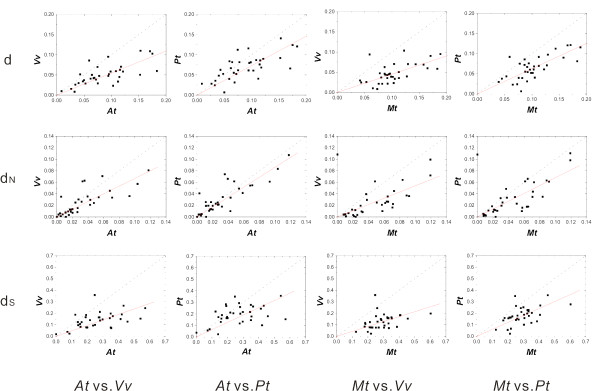
**Scatter plots of evolutionary rate in annuals against that in perennials for chloroplast genes estimated by the ML method**. Cases in all 4 annual-perennial cross-comparison are shown. The dotted line is the diagonal line with a slope equal to 1, and the red line is the regression line.

Among the 34 chloroplast genes, 64.7-97.1% showed higher evolutionary rates in annuals than in perennials in our cross-comparisons (Table 1). The sign-test and the paired *t*-test clearly showed heterogeneity in the rates of molecular evolution between annuals and perennials (Table 1 and 2). Moreover, the regression slopes in the scatter plots were smaller than unity (0.541-0.730) between annuals and perennials in all the 12 comparison pairs (Figure 4 and Table 3). For chloroplast genes, the correlation between annual-perennial evolutionary rates in *A. thaliana *appeared to be still detectable after synonymous substitutions were incorporated (R^2 ^= 0.358-0.531 for d) (Table 3). The overall range of d_S _in the 34 chloroplast genes calculated for *A. thaliana *was 0-0.57 with an average of 0.27 (SD = 0.13), suggesting a minimal saturation effect on the estimation of evolutionary rate in the chloroplast genes.

## Discussion

### Possible mechanisms for the higher evolutionary rate in annuals

In this study, we examined a large number of gene loci across both nuclear and chloroplast genomes in four model plants. These genes encode proteins with diverse functions in plants, including structural proteins, metabolic enzymes, and transcription factors. Therefore, they represent genes with different functional constraints well, and they can be used as a proxy for inferring the situation across the whole plant genome. In nuclear genomes, our survey on 85 nuclear housekeeping genes and 10 gene families consistently revealed a generally higher evolutionary rate in annuals than in perennials. Similar pattern was found in chloroplast genomes in this study, which is consistent with a previous study based on two chloroplast genes [22]. In addition, consistent results have been reported at several loci in mitochondrial genes, e.g. in *atp*A, *cox*I, and some non-coding regions [12,23-25]. Altogether, the consistent results from various genomes suggest a globally faster evolutionary tempo at the molecular level for annuals than for perennials across both nuclear and organelle genomes.

Following this line of reasoning, such a global pattern should be shaped by some organism-level factors which influence both nuclear and organelle genomes. Generation-time might be one of such factors. Assuming that mutations occur predominantly at cell division and that the spontaneous meiotic and mitotic mutation rate per cell cycle are the same for annual and perennial plants, the observed difference in evolutionary rate should depend on the frequency of cell division per unit time. Since annual plants take a shorter time from germination to reach first flowering than perennials, they might on average experience more frequent cell divisions per unit time prior to reproduction. If this is true, a generally faster evolutionary tempo in annuals than in perennials, as observed in our results, is expected. Meanwhile, our results suggest that the lifelong accumulated somatic mutations, mostly introduced by mitosis, have not offset the generation time effect on annual-perennial evolutionary rate difference, which is consistent with other studies [15].

While generation-time might at least partly account for the evolutionary rate heterogeneity between annuals and perennials, there are other possibilities to generate the observed pattern. For example, the observed interspecific rate heterogeneity could also be attributed to the difference in their effective population size (Ne) [26]. Ne is an important factor that influences the balance among mutation, random genetic drift and natural selection. As predicted by the Nearly Neutral Mutation Model [9,27], smaller Ne leads to higher probability of fixation of mutations by random genetic drift, and hence higher evolutionary rate. In plants, there are strong association between outcrossing rate and the perenniality of the species [28], and an annual species tend to be a selfing species and has generally lower Ne compared with perennials [15,29-31]. And the observation of strongly negative selection for inbreeding in perennial plants further enforced the tendency of a genome-wide lower evolutionary rate in perennials than in their annual counterparts [32].

Different speciation rate may be also an alternative explanation. It has been shown that perennials appear to have slower speciation rates than annuals [33]. Thus, the lower frequency of speciation events in perennials and fewer corresponding opportunities for bottleneck and adaptation events may result in lower evolutionary rates, especially at nonsynonymous sites, compared with annuals [4]. In addition, the slower metabolic rate and fewer recombination events per unit time in perennials might also result in the higher spontaneous mutation rate per unit time in annuals [34]. Therefore, while the difference in evolutionary tempo between annuals and perennials seems to be quite pronounced, the biological mechanism behind this phenomenon is still unclear.

### Pronounced evolutionary rate correlation among species

Another interesting finding was clearly linear correlations of evolutionary rates at the nonsynonymous sites. The square of correlation coefficient (R^2^) ranges from 0.280-0.593 in nuclear genome and 0.297-0.721 in chloroplast genome. Moreover, in contrast to the fast decay of correlation at synonymous sites, the strong correlation at nonsynonymous sites held quite well even between the highly diverged taxa. Therefore, it seems that the relative evolutionary rate between two compared species was relatively constant across multiple gene loci in plant genomes, showing an analogous clock-behavior. This observation reminds us a classic concept of molecular clock, which claims an approximately constant evolutionary rate over time for any given protein or DNA sequence in all lineages [35]. Herein, the roughly constant relative rate of nonsynonymous substitutions among species at the genomic level might provide a robust measure to calibrate the lineage effect of the local molecular clock.

### Exception genes against the global trend of higher evolutionary rate in annuals

Nine nuclear housekeeping genes and one chloroplast gene were identified due to their consistently higher rate of molecular evolution in perennials rather than in annuals in all four cross-comparisons, based on one or more estimators (Table S10 in Additional file 1). Among them, Transketolase, Prenylcysteine oxidase 1 and PsaC are the most noticeable, showing consistently higher rates of evolution in perennials among d, d_N _and d_S_. Transketolase is an enzyme that participates in the transfer of ketol groups and catalyzes three reactions in the regenerative phase of the Calvin cycle [36]. It also participates in non-oxidative branch of the pentose phosphate pathway and Rubisco shunt. Prenylcysteine oxidase 1 is involved in the farnesyl diphosphate metabolic process, and plays an important role in detoxification and recycling of farnesylcysteine [37]. In plant chlorplast, PsaC is an important subunit of photosystem I (PSI) which provides the ligands for the terminal electron acceptors, F_A _and F_B _[38,39]. Such pronounced disagreements with the global trend of faster evolution tempo in annuals together with the critical biological functions of these two genes hint the effect of selective force at least at the species-specific level. It will be interesting to test whether such exceptions hold the same in the annual-perennial comparisons of other species.

### The limitations of this study

We acknowledge that the selected species in this study were not ideal by two reasons. Firstly, their phylogenetic relationships were too distant to avoid the saturation effect (Figure 1, 2). This was inevitable for the genomic survey due to the limited availability of genomic information at this moment, but comparisons between more closely related pairs of annual and perennial species would have provided a better resolution without suffering from the saturation effect. Sibling species comparisons have been used in plant biology in different contexts (e.g., Gitzendanner and Soltis [40]) and proven to be fruitful. Nevertheless, we believe that our conclusions on the heterogeneity of evolutionary rate between annuals and perennials are valid because the lack of resolution due to saturation effect generally makes the results conservative. Secondly, our sample size (four species plus one outgroup) was restricted, and the comparisons were non-independent (two annuals by two perennials). This is also inevitable at this moment, and we should not over-generalize our conclusions to non-model species. Since more and more genomic information is becoming available, future studies on new genomic data of additional close-related plants and deeper understanding of their life histories, including annuals and perennials, will soon provide further knowledge about the generality and the mechanism of heterogeneous evolutionary rate between these species.

## Conclusions

Four annual-perennial comparisons based on multiple gene loci and the combination of nuclear and chloroplast genomes consistently suggested higher evolutionary rates in annual plants. Together with previous studies, we propose that the difference in evolutionary rate between annuals and perennials is a genome-wide phenomenon in plants, thereby shaped by some genome-wide factors associated with their annual/perennial life histories. Besides, it is noteworthy that nucleotide substitution rate at nonsynonymous sites appears to correlate well among compared species in our study, implying a roughly constant relative rate of molecular evolution across different gene loci. Finally, a few consistent exceptions of the global trends of faster evolution tempo in annuals were observed in all four cross-comparisons of our study, indicating noticeable effects of natural selection in these gene loci. While we acknowledge the methodological limitations of this study based on a small number of distantly-related model species, our results indicate that the genome-wide comparison is a promising approach to further understanding the mechanism determining the molecular evolutionary rate at the genomic level in plants.

## Methods

### Data sampling

Five different plant taxa were investigated in this study, including four dicots (*Arabidopsis thaliana*, *Medicago truncatula*, *Vitis vinifera*, and *Populus trichocarpa*) and one monocot (*Oryza sativa*). Their nuclear genome sequences and annotation data were downloaded from online databases; the detailed download websites and the data version can be found in Table S11 (Additional file 1). The corresponding chloroplast genomes data were downloaded from Genbank. The evolutionary history of these five species was inferred based on multi-gene matrix as described below [41,42].

Eighty-five housekeeping genes were randomly selected, based on the enzymes encoded by housekeeping genes in *A. thaliana *[43] and very conserved genes throughout plant evolution from moss to flowering plants [44] (Table S12 in Additional file 1). 34 chloroplast genes were randomly chosen according to two criteria: 1) the gene is conserved across the five different plant species used in our study, 2) the length of the gene should be >200 base pairs (bp) to give enough substitution information (Table S13 in Additional file 1). 10 non-housekeeping gene families with a wide spectrum of homolog gene numbers and functional constraints were randomly sampled according to the gene family list on TAIR and Pfam database v23.0 (Table S14 in Additional file 1).

Amino acid sequences of the 85 nuclear housekeeping genes were first identified using BLASTP in each plant genome. Then, the corresponding nucleotide sequences of the CDS regions were obtained and employed in further analysis. Both amino acid and nucleotide coding sequences of the 34 chloroplast genes in each of the five studied species were identified according to Gramene.

For non-housekeeping gene families, both BLAST and hidden Markov model (HMM) searches were performed to identify the homologous genes of every gene family in each species. Firstly, the amino acid consensus sequence of the representative domain of each gene family was retrieved from the Pfam database and was adopted as the query in BLASTP searches for all possible homologs encoded in our sampled genomes. The threshold of expectation value was set to 1.0, a value determined empirically to filter out most spurious hits. Next, all candidate hits in each gene family were examined to further verify whether they encoded the representative motif of this specific family using hmmpfam based on the Pfam database with an E value cut-off of 10^-4^. Using this method, all homolog genes for each of the 10 non-housekeeping gene families in our sampled five plant genomes were identified. Then, the phylogenetic tree was reconstructed for each gene family based on the neighbor-joining (NJ) method. Gene families usually experiences extensive segmental and tandem duplication over the evolutionary history, thus bringing difficulties in identifying their orthologous relationships across different species. Therefore, only those monophyletic clades which cover all four dicots and also have > 50 bootstrap values were sampled in this study. The neighboring homologous genes surround the sampled clade were used as a proxy of outgroup. Although this sampling criterion is relatively strict, generally > 50% members were sampled for each gene family. Following this strategy, our total dataset of 111 orthologous clades were sampled from those 10 non-housekeeping gene families. Since the number of clades sampled in each gene families varies considerably, simply using this total dataset to conduct statistic analysis may introduce latent sampling bias. Therefore, this total dataset was further divided into three sub-datasets. Sub-dataset 1 contains 35 clades which sampled from eight relatively small gene families. Sub-dataset 2 is comprised of 24 clades, all sampled from PP2C gene family. Finally, sub-dataset 3 covers 52 clades and represents LRR-Pkinase gene family. Further analyses were conducted for not only the total dataset but all these three sub-datasets as well.

### Sequence alignment and phylogenetic reconstruction

Phylogenetic reconstruction was performed for both revealing evolutionary relationship among our sampled five species and estimating evolutionary rate in each gene locus or clade. The sequences of those 34 chloroplast genes and 85 housekeeping genes were respectively concatenated in unique matrix to infer the evolutionary history of *O. sativa*, *A. thaliana*, *M. truncatula*, *V. vinifera *and *P. trichocarpa*. The amino acid sequences of this matrix were aligned by ClustalW with default options [45] and the resulting alignments were then used to guide the alignments of nucleotide coding sequences (CDSs). The phylogenetic tree was constructed based on the neighbor-joining (NJ) method with the Kimura's 2-parameter model using MEGA program v4.0 [46]. All positions containing gaps or missing data were eliminated from the dataset (Complete deletion option in MEGA). The stability of internal nodes was assessed by bootstrap analysis with 1,000 replicates. For each of our sampled individual gene loci or clades, the phylogenetic tree was also reconstructed independently following similar procedures. The tree topologies of these individual gene loci or clades were then used as input files for PAML package (v4.4) [47] in molecular evolution estimation. We also used Phylip program version 3.69 [48] with the Maximum Likelihood (ML) method to confirm the phylogenetic relationship, and obtained the same topology as NJ trees for nuclear genes with 100% bootstrap supports (data not shown). For chloroplast genes, the ML consensus tree suggested a different topology (*P. trichocarpa *clustered with *M. truncatula *before the clustering with *V. vinifera*). The bootstrap support for the clustering of *P. trichocarpa *and *M. truncatula *was rather low (50.6%) in this case, however. Thus, we present only the results from the NJ method (Figure 1).

### Evolutionary rate estimation

Theoretically, for each of our sampled gene loci or clades, the evolutionary rates of two compared species A and B after they have diverged from their common ancestor with reference to the outgroup species O, can be estimated by the equation d_A _= (d_AB_+d_AO_-d_BO_)/2 and d_B _= (d_AB_+d_BO_-d_AO_)/2 respectively [23,49]. In our study, four annual-perennial pairs, *At *vs *Vv*, *At *vs *Pt*, *Mt *vs *Vv*, *Mt *vs *Pt*, were compared, with *Oryza sativa *as an outgroup. For species with multiple copies of paralogs, its evolutionary rate was estimated by calculating the mathematic mean of those paralogs. A total of three different measurements, the nucleotide substitution number per site (d), the proportion of nonsynonymous difference (p_N_) and the proportion of synonymous difference (p_S_), were employed to estimate evolutionary rates. d was calculated using the Kimura's 2-parameter method and p_N _and p_S _were calculated using the Nei-Gojobori method by MEGA.

In addition, we used the maximum likelihood (ML) method to compare the evolutionary rates. Since this method does not necessarily depend on the sequence information from the outgroup, which is largely distant from the dicot species in our study (Figure 1), the ML method might minimize the saturation effect and provide better estimates. As in Ronald et al. [50], we implemented the branch-specific likelihood model with the codeml program in PAML 4.4 in our calculation. The branch models allow different d_N_/d_S _for different branches along the phylogeny. The ML branch lengths from tips of the two compared species to their nearest node were collected and compared for each annual-perennial pair. For species with multiple copies of paralogs, their evolutionary rate was estimated by calculating the mathematic mean of those paralogs. Three different estimators, nucleotide substitution rate per site (d), nonsynonymous substitution rate per site (d_N_), and synonymous substitution rate per site (d_S_), were employed to estimate evolutionary rates.

Both the outgroup-dependent method and the ML method were used in this study and generally consistent results were obtained. Therefore, only results from the ML method were shown in the text for simplicity. The results from the outgroup-dependent method were attached in Additional files 2 and 1 (Figure S1-S2 and Table S1-S6).

### Statistical analysis

The differences between annual and perennial evolutionary rates were calculated for each comparison pair and both sign-test and paired *t*-test were used to assess the null hypothesis of equal evolutionary rates between annuals and perennials. According to the sign-test, if the ratio of plus signs to minus signs deviates from the expected 1:1 ratio significantly, the null hypothesis is rejected, suggesting different evolutionary rates in annuals and perennials. Paired-*t *test, on the other hand, measures whether the means of the same subject from the two compared groups vary significantly from each other, thus taking into account the degree of difference in each matched pair.

The correlation analysis was conducted using Origin software. Following the biological hypothesis that two compared species diverge from a common ancestor, if their evolutionary rates do correlate, the regression line should pass through the origin. Thus, the square of correlation coefficient (R^2^) is calculated under this assumption.

## Abbreviations

CDS: Coding sequence; DNA: Deoxyribonucleic acid; ML: Maximum likelihood; PP2C: Protein Phosphatase 2C; LRR: Leucine Rich Repeat; TAIR: The Arabidopsis Information Resource; BLAST: The Basic Local Alignment Search Tool; HMM: Hidden Markov models;

## Authors' contributions

DT, SY and JXY designed the research, JXY, JL and DW performed the research and the analyses; JXY and SY wrote the manuscript. HA, and DT helped with the discussion and worked on the manuscript. All authors read and approved the final manuscript.

## Supplementary Material

Additional file 1 **Supplemental Tables**. Including all supplemental tables. **Table S1**. Sign-test for the annual-perennial comparison of evolutionary rates, estimated by the outgroup-dependent method. The proportion of genes showing higher evolutionary rate in annuals than in perennials and the *P*-value of sign-test in three different measures of the evolutionary rate (d, p_N _and p_S_) are listed in the table. **Table S2**. *P*-values by the paired *t*-test for the heterogeneity of evolutionary rates. The *P*-values of paired *t*-test in all 4 annual-perennial cross-comparisons suggest higher evolutionary rates in annuals than in perennials. The estimation of evolutionary rate is based on the outgroup-dependent method. **Table S3**. Correlations between annual-perennial evolutionary rates based on the outgroup-dependent method. The square of correlation coefficient (R^2^) and the slope of regression line of evolutionary rate in annuals against that in perennials are listed in the table. **Table S4**. Sign-test for annual-perennial comparison of evolutionary rates, estimated by the outgroup-dependent method, for the 3 sub-datasets sampled from non-housekeeping gene families. The *P*-value of sign-test in all 4 annual-perennial cross-comparisons and the proportion of genes showing higher evolutionary rate in annuals than in perennials are listed in the table. **Table S5**. Paired *t*-test for annual-perennial comparison of evolutionary rates, estimated by the outgroup-dependent method, for the 3 sub-datasets sampled from non-housekeeping gene families. The *P*-value of paired *t*-test in all 4 annual-perennial cross-comparisons show higher evolutionary rate in annuals than in perennials. **Table S6**. Correlation between annual-perennial evolutionary rates estimated by the outgroup-dependent method for the 3 sub-datasets sampled from non-housekeeping gene families. The square of correlation coefficient (R^2^) and the slope of regression line of evolutionary rate in annuals against that in perennials are listed in the table. **Table S7**. Sign-test for annual-perennial comparison of evolutionary rates, estimated by the ML method, for the 3 sub-datasets sampled from non-housekeeping gene families. The *P*-value of sign-test in all 4 annual-perennial cross-comparisons and the proportion of genes showing higher evolutionary rate in annuals than in perennials are listed in the table. **Table S8**. Paired *t*-test for annual-perennial comparison of evolutionary rates, estimated by the ML method, in the 3 sub-datasets sampled from non-housekeeping gene families. The *P*-value of sign-test in all 4 annual-perennial cross-comparisons show higher evolutionary rate in annuals than in perennials. **Table S9**. Correlation between annual-perennial evolutionary rates, estimated by the ML method, for the 3 sub-datasets sampled from non-housekeeping gene families based on the data estimated by the ML method. The square of correlation coefficient (R^2^) and the slope of regression line of evolutionary rate in annuals against that in perennials are listed in the table. **Table S10**. Exception genes against the global trend of higher evolutionary rates in annuals than in perennials. The exception here is defined as showing consistent disagreements with the global trends of faster evolution tempo in annuals than in perennials in all 4 annual-perennial cross comparisons. **Table S11**. Plant genome data used in this study. **Table S12**. Housekeeping genes sampled in this study **Table S13**. Chloroplast genes sampled in this study. **Table S14**. Non-housekeeping gene families sampled in this study. Click here for file

Additional file 2 **Supplemental Figures**. Including all supplemental figures. **Figure S1**. Scatter plots of evolutionary rate of annuals against that of perennials for both nuclear and chloroplast genes estimated by the outgroup-dependent method. Cases in all 4 annual-perennial cross-comparison are shown. The dash line is the diagonal line with a slope equals to 1, and the red line is the regression line. **Figure S2**. Scatter plots of evolutionary rates of annuals against that of perennials for all 3 sub-datasets of non-housekeeping gene families estimated by the outgroup-dependent method. Cases in all 4 annual-perennial cross-comparison are shown. The dash line is the diagonal line with a slope equals to 1, and the red line is the regression line **Figure S3**. Scatter plots of evolutionary rate in annuals against that in perennials for the 3 sub-datasets collected from non-housekeeping gene families estimated by the ML method. Cases in all 4 annual-perennial cross-comparisons are shown. The dash line is the diagonal line with a slope equals to 1, and the red line is the regression line Click here for file
