# Supplementary material for: Genome-wide investigation reveals high evolutionary rates in annual model plants
Source: BMC Plant Biol. 2010 Nov 9;10:242. doi: 10.1186/1471-2229-10-242 (PMC3095324; doi:10.1186/1471-2229-10-242)
Supplement: Additional file 1 — Supplemental Tables. Including all supplemental tables. Table S1. Sign-test for the annual-perennial comparison of evolutionary rates, estimated by the outgroup-dependent method. The proportion of genes showing higher evolutionary rate in annuals than in perennials and the P-value of sign-test in three different measures of the evolutionary rate (d, pN and pS) are listed in the table. Table S2. P-values by the paired t-test for the heterogeneity of evolutionary rates. The P-values of paired t-test in all 4 annual-perennial cross-comparisons suggest higher evolutionary rates in annuals than in perennials. The estimation of evolutionary rate is based on the outgroup-dependent method. Table S3. Correlations between annual-perennial evolutionary rates based on the outgroup-dependent method. The square of correlation coefficient (R2) and the slope of regression line of evolutionary rate in annuals against that in perennials are listed in the table. Table S4. Sign-test for annual-perennial comparison of evolutionary rates, estimated by the outgroup-dependent method, for the 3 sub-datasets sampled from non-housekeeping gene families. The P-value of sign-test in all 4 annual-perennial cross-comparisons and the proportion of genes showing higher evolutionary rate in annuals than in perennials are listed in the table. Table S5. Paired t-test for annual-perennial comparison of evolutionary rates, estimated by the outgroup-dependent method, for the 3 sub-datasets sampled from non-housekeeping gene families. The P-value of paired t-test in all 4 annual-perennial cross-comparisons show higher evolutionary rate in annuals than in perennials. Table S6. Correlation between annual-perennial evolutionary rates estimated by the outgroup-dependent method for the 3 sub-datasets sampled from non-housekeeping gene families. The square of correlation coefficient (R2) and the slope of regression line of evolutionary rate in annuals against that in perennials are listed in the table. Table S7 [file 1471-2229-10-242-S1.DOC]

**Table S1.** Sign-test for the annual-perennial comparison of evolutionary rates, estimated by the outgroup-dependent method. The proportion of genes showing higher evolutionary rate in annuals than in perennials and the *P*-value of sign-test in three different measures of the evolutionary rate (d, p_N_ and p_S_) are listed in the table.

|  | | | | | | | | | | | | | | |
| --- | --- | --- | --- | --- | --- | --- | --- | --- | --- | --- | --- | --- | --- | --- |
| **Nuclear Housekeeping Genes (85 Loci)** | | | | | | | | | | | | | | |
| **Comparison**  **Pair** |  | **d** | |  | **p_N_** | | | |  | | **p_S_** | | | |
|  |  | **Proportion** | **P-Value** |  | **Proportion** | | **P-Value** | |  | | **Proportion** | | **P-Value** | |
| ***At* vs. *Vv*** |  | 90.6% | 2.77E-15 |  | 88.2% | | 1.86E-13 | |  | | 76.5% | | 4.75E-07 | |
| ***At* vs. *Pt*** |  | 76.5% | 1.03E-06 |  | 80.0% | | 8.54E-09 | |  | | 63.5% | | 0.017 | |
| ***Mt* vs. *Vv*** |  | 85.9% | 8.09E-12 |  | 76.5% | | 2.11E-07 | |  | | 81.2% | | 5.24E-09 | |
| ***Mt* vs. *Pt*** |  | 69.4% | 4.47E-04 |  | 70.6% | | 1.07E-04 | |  | | 62.4% | | 0.029 | |
|  | | | | | | | | | | | | | | |
| **Non-housekeeping Gene Families (111 Clades)** | | | | | | | | | | | | | | |
| **Comparison**  **Pair** |  | **d** | |  | **p_N_** | | | |  | | **p_S_** | | | |
|  |  | **Proportion** | **P-Value** |  | **Proportion** | | **P-Value** | |  | | **Proportion** | | **P-Value** | |
| ***At* vs. *Vv*** |  | 87.4% | 1.89E-16 |  | 89.2% | | 3.45E-18 | |  | | 72.1% | | 3.68E-06 | |
| ***At* vs. *Pt*** |  | 77.5% | 9.59E-10 |  | 89.2% | | 7.37E-19 | |  | | 54.1% | | 0.391 | |
| ***Mt* vs. *Vv*** |  | 80.2% | 9.63E-11 |  | 80.2% | | 9.63E-11 | |  | | 75.7% | | 1.22E-08 | |
| ***Mt* vs. *Pt*** |  | 76.6% | 1.70E-08 |  | 75.7% | | 5.46E-08 | |  | | 73.0% | | 1.37E-06 | |
|  |  |  |  |  | |  | |  | |  | |  | |  |
| **Chloroplast Genes (34 Loci)** | | | | | | | | | | | | | | |
| **Comparison**  **Pair** |  | **d** | |  | **p_N_** | | | |  | | **p_S_** | | | |
|  |  | **Proportion** | **P-Value** |  | **Proportion** | | **P-Value** | |  | | **Proportion** | | **P-Value** | |
| ***At vs. Vv*** |  | 85.3% | 1.10E-05 |  | 79.4% | | 1.13E-04 | |  | | 88.2% | | 6.16E-06 | |
| ***At vs. Pt*** |  | 67.7% | 0.058 |  | 67.7% | | 0.011 | |  | | 76.5% | | 2.94E-03 | |
| ***Mt vs. Vv*** |  | 94.1% | 6.94E-08 |  | 91.2% | | 7.66E-07 | |  | | 94.1% | | 6.94E-08 | |
| ***Mt vs. Pt*** |  | 82.4% | 1.90E-05 |  | 82.4% | | 6.60E-05 | |  | | 85.3% | | 3.90E-05 | |

**Table S2.** *P*-values by the paired *t*-test for the heterogeneity of evolutionary rates. The *P*-values of paired *t*-test in all 4 annual-perennial cross-comparisons suggest higher evolutionary rates in annuals than in perennials. The estimation of evolutionary rate is based on the outgroup-dependent method.

|  | | | | | | | | | | | | | | |
| --- | --- | --- | --- | --- | --- | --- | --- | --- | --- | --- | --- | --- | --- | --- |
| **Nuclear Housekeeping Genes (85 Loci)** | | | | | | | | | | | | | | |
| **Comparison**  **Pair** |  | **d** | |  | **p_N_** | | | |  | | **p_S_** | | | |
|  |  | ***Vv*** | ***Pt*** |  | ***Vv*** | | ***Pt*** | |  | | ***Vv*** | | ***Pt*** | |
| ***At*** |  | 4.81E-17 | 3.09E-08 |  | 1.05E-11 | | 2.52E-10 | |  | | 1.19E-08 | | 0.228 | |
| ***Mt*** |  | 8.48E-11 | 9.30E-04 |  | 6.08E-06 | | 5.62E-04 | |  | | 4.67E-09 | | 0.061 | |
|  | | | | | | | | | | | | | | |
| **Non-housekeeping Gene Families (111 Clades)** | | | | | | | | | | | | | | |
| **Comparison**  **Pair** |  | **d** | |  | **p_N_** | | | |  | | **p_S_** | | | |
|  |  | ***Vv*** | ***Pt*** |  | ***Vv*** | | ***Pt*** | |  | | ***Vv*** | | ***Pt*** | |
| ***At*** |  | 2.1E-18 | 7.69E-08 |  | 3.61E-17 | | 3.15E-14 | |  | | 1.81E-04 | | 0.764 | |
| ***Mt*** |  | 6.27E-15 | 3.33E-09 |  | 9.35E-11 | | 1.28E-08 | |  | | 1.53E-11 | | 8.16E-07 | |
|  |  |  |  |  | |  | |  | |  | |  | |  |
| **Chloroplast Genes (34 Loci)** | | | | | | | | | | | | | | |
| **Comparison**  **Pair** |  | **d** | |  | **p_N_** | | | |  | | **p_S_** | | | |
|  |  | ***Vv*** | ***Pt*** |  | ***Vv*** | | ***Pt*** | |  | | ***Vv*** | | ***Pt*** | |
| ***At*** |  | 1.32E-07 | 3.46E-03 |  | 4.48E-05 | | 0.0421 | |  | | 1.93E-08 | | 3.28E-03 | |
| ***Mt*** |  | 9.28E-12 | 1.54E-06 |  | 4.22E-08 | | 1.75E-06 | |  | | 9.27E-10 | | 7.54E-04 | |

**Table S3.** Correlation between annual-perennial evolutionary rates based on the outgroup-dependent method. The square of correlation coefficient (R^2^) and the slope of regression line of evolutionary rate in annuals against that in perennials are listed in the table.

|  | | | | | | | | | | | | | | | |
| --- | --- | --- | --- | --- | --- | --- | --- | --- | --- | --- | --- | --- | --- | --- | --- |
| **Nuclear Housekeeping Genes (85 Loci)** | | | | | | | | | | | | | | | |
| **Comparison**  **Pair** |  | **d** | |  | **p_N_** | | | |  | | **p_S_** | | | |  |
|  |  | **R^2^** | **Slope** |  | **R^2^** | | **Slope** | |  | | **R^2^** | | **Slope** | |  |
| ***At vs. Vv*** |  | 0.203 | 0.721 |  | 0.423 | | 0.751 | |  | | 0.018 | | 0.857 | |  |
| ***At vs. Pt*** |  | 0.183 | 0.805 |  | 0.471 | | 0.776 | |  | | 0.002 | | 0.969 | |  |
| ***Mt vs. Vv*** |  | 0.111 | 0.714 |  | 0.366 | | 0.795 | |  | | 0.001 | | 0.830 | |  |
| ***Mt vs. Pt*** |  | 0.012 | 0.819 |  | 0.277 | | 0.830 | |  | | 0.044 | | 0.963 | |  |
|  | | | | | | | | | | | | | | | |
| **Non-housekeeping Gene Families (111 Clades)** | | | | | | | | | | | | | | | |
| **Comparison**  **Pair** |  | **d** | |  | **p_N_** | | | |  | | **p_S_** | | | |  |
|  |  | **R^2^** | **Slope** |  | **R^2^** | | **Slope** | |  | | **R^2^** | | **Slope** | |  |
| ***At vs. Vv*** |  | 0.311 | 0.768 |  | 0.539 | | 0.798 | |  | | 0.009 | | 0.941 | |  |
| ***At vs. Pt*** |  | 0.201 | 0.829 |  | 0.558 | | 0.819 | |  | | 0.001 | | 0.998 | |  |
| ***Mt vs. Vv*** |  | 0.122 | 0.682 |  | 0.517 | | 0.811 | |  | | 0.013 | | 0.865 | |  |
| ***Mt vs. Pt*** |  | 0.158 | 0.767 |  | 0.573 | | 0.844 | |  | | 0.051 | | 0.930 | |  |
|  |  |  |  |  | |  | |  | |  | |  | |  | |
| **Chloroplast Genes (34 Loci)** | | | | | | | | | | | | | | | |
| **Comparison**  **Pair** |  | **d** | |  | **p_N_** | | | |  | | **p_S_** | | | |  |
|  |  | **R^2^** | **Slope** |  | **R^2^** | | **Slope** | |  | | **R^2^** | | **Slope** | |  |
| ***At vs. Vv*** |  | 0.473 | 0.576 |  | 0.643 | | 0.607 | |  | | 0.452 | | 0.676 | |  |
| ***At vs. Pt*** |  | 0.587 | 0.816 |  | 0.814 | | 0.825 | |  | | 0.356 | | 0.848 | |  |
| ***Mt vs. Vv*** |  | 0.426 | 0.528 |  | 0.645 | | 0.545 | |  | | 0.068 | | 0.635 | |  |
| ***Mt vs. Pt*** |  | 0.456 | 0.710 |  | 0.823 | | 0.718 | |  | | 0.001 | | 0.773 | |  |

**Table S4.** Sign-test for annual-perennial comparison of evolutionary rates, estimated by the outgroup-dependent method, for the 3 sub-datasets sampled from non-housekeeping gene families. The *P*-value of sign-test in all 4 annual-perennial cross-comparisons and the proportion of genes showing higher evolutionary rate in annuals than in perennials are listed in the table.

|  | | | | | | | | | | | | | | |
| --- | --- | --- | --- | --- | --- | --- | --- | --- | --- | --- | --- | --- | --- | --- |
| **Sub-dataset 1: 8 different gene families (35 Clades)** | | | | | | | | | | | | | | |
| **Comparison**  **Pair** |  | **d** | |  | **p_N_** | | | |  | | **p_S_** | | | |
|  |  | **Proportion** | **P-Value** |  | **Proportion** | | **P-Value** | |  | | **Proportion** | | **P-Value** | |
| ***At* vs. *Vv*** |  | 77.14% | 1.88E-03 |  | 82.86% | | 1.17E-04 | |  | | 60.00% | | 0.311 | |
| ***At* vs. *Pt*** |  | 74.29% | 5.99E-03 |  | 80.00% | | 1.95E-04 | |  | | 54.29% | | 0.736 | |
| ***Mt* vs. *Vv*** |  | 74.29% | 5.99E-03 |  | 80.00% | | 5.08E-04 | |  | | 74.29% | | 5.99E-03 | |
| ***Mt* vs. *Pt*** |  | 77.14% | 1.88E-03 |  | 80.00% | | 5.08E-04 | |  | | 71.43% | | 0.017 | |
|  | | | | | | | | | | | | | | |
| **Sub-dataset 2: PP2C gene family (24 Clades)** | | | | | | | | | | | | | | |
| **Comparison**  **Pair** |  | **d** | |  | **p_N_** | | | |  | | **p_S_** | | | |
|  |  | **Proportion** | **P-Value** |  | **Proportion** | | **P-Value** | |  | | **Proportion** | | **P-Value** | |
| ***At* vs. *Vv*** |  | 87.50% | 2.77E-04 |  | 83.33% | | 1.54E-03 | |  | | 75.00% | | 0.023 | |
| ***At* vs. *Pt*** |  | 87.50% | 2.77E-04 |  | 95.83% | | 2.98E-06 | |  | | 54.17% | | 0.678 | |
| ***Mt* vs. *Vv*** |  | 79.17% | 6.61E-03 |  | 66.67% | | 0.152 | |  | | 70.83% | | 0.035 | |
| ***Mt* vs. *Pt*** |  | 79.17% | 6.61E-03 |  | 75.00% | | 0.023 | |  | | 66.67% | | 0.152 | |
|  |  |  |  |  | |  | |  | |  | |  | |  |
| **Sub-dataset 3: LRR-Pkinase gene family** **(52 Clades)** | | | | | | | | | | | | | | |
| **Comparison**  **Pair** |  | **d** | |  | **p_N_** | | | |  | | **p_S_** | | | |
|  |  | **Proportion** | **P-Value** |  | **Proportion** | | **P-Value** | |  | | **Proportion** | | **P-Value** | |
| ***At* vs. *Vv*** |  | 94.23% | 1.04E-11 |  | 96.15% | | 6.12E-13 | |  | | 78.85% | | 3.60E-05 | |
| ***At* vs. *Pt*** |  | 75.00% | 9.00E-05 |  | 92.31% | | 1.31E-10 | |  | | 53.85% | | 0.678 | |
| ***Mt* vs. *Vv*** |  | 84.62% | 4.04E-07 |  | 86.54% | | 6.97E-08 | |  | | 78.85% | | 1.50E-05 | |
| ***Mt* vs. *Pt*** |  | 75.00% | 4.10E-04 |  | 73.08% | | 1.20E-03 | |  | | 76.92% | | 1.28E-04 | |

**Table S5.** Paired *t*-test for annual-perennial comparison of evolutionary rates, estimated by the outgroup-dependent method, for the 3 sub-datasets sampled from non-housekeeping gene families. The *P*-value of paired *t*-test in all 4 annual-perennial cross-comparisons show higher evolutionary rate in annuals than in perennials.

|  | | | | | | | | | | | | | | |
| --- | --- | --- | --- | --- | --- | --- | --- | --- | --- | --- | --- | --- | --- | --- |
| **Sub-dataset 1: 8 different gene families (35 Clades)** | | | | | | | | | | | | | | |
| **Comparison**  **Pair** |  | **d** | |  | **p_N_** | | | |  | | **p_S_** | | | |
|  |  | ***Vv*** | ***Pt*** |  | ***Vv*** | | ***Pt*** | |  | | ***Vv*** | | ***Pt*** | |
| ***At*** |  | 1.95E-04 | 0.011 |  | 3.10E-04 | | 2.77E-04 | |  | | 0.369 | | 0.359 | |
| ***Mt*** |  | 1.76E-04 | 2.89E-03 |  | 5.90E-03 | | 1.66E-03 | |  | | 7.55E-04 | | 7.94E-03 | |
|  | | | | | | | | | | | | | | |
| **Sub-dataset 2: PP2C gene family (24 Clades)** | | | | | | | | | | | | | | |
| **Comparison**  **Pair** |  | **d** | |  | **p_N_** | | | |  | | **p_S_** | | | |
|  |  | ***Vv*** | ***Pt*** |  | ***Vv*** | | ***Pt*** | |  | | ***Vv*** | | ***Pt*** | |
| ***At*** |  | 2.02E-06 | 1.88E-04 |  | 1.36E-06 | | 1.99E-07 | |  | | 0.027 | | 0.369 | |
| ***Mt*** |  | 4.82E-04 | 2.05E-03 |  | 0.016 | | 8.90E-03 | |  | | 6.36E-03 | | 0.011 | |
|  |  |  |  |  | |  | |  | |  | |  | |  |
| **Sub-dataset 3: LRR-Pkinase gene family (52 Clades)** | | | | | | | | | | | | | | |
| **Comparison**  **Pair** |  | **d** | |  | **p_N_** | | | |  | | **p_S_** | | | |
|  |  | ***Vv*** | ***Pt*** |  | ***Vv*** | | ***Pt*** | |  | | ***Vv*** | | ***Pt*** | |
| ***At*** |  | 1.7E-16 | 1.53E-06 |  | 1.03E-15 | | 1.05E-10 | |  | | 6.92E-06 | | 0.746 | |
| ***Mt*** |  | 1.86E-10 | 2.50E-06 |  | 3.22E-11 | | 2.24E-05 | |  | | 2.30E-08 | | 4.08E-06 | |

**Table S6.** Correlation between annual-perennial evolutionary rates estimated by the outgroup-dependent method for the 3 sub-datasets sampled from non-housekeeping gene families. The square of correlation coefficient (R^2^) and the slope of regression line of evolutionary rate in annuals against that in perennials are listed in the table.

|  | | | | | | | | | | | | | | |
| --- | --- | --- | --- | --- | --- | --- | --- | --- | --- | --- | --- | --- | --- | --- |
| **Sub-dataset 1: 8 different gene families (35 Clades)** | | | | | | | | | | | | | | |
| **Comparison**  **Pair** |  | **d** | |  | **p_N_** | | | |  | | **p_S_** | | | |
|  |  | **R^2^** | **Slope** |  | **R^2^** | | **Slope** | |  | | **R^2^** | | **Slope** | |
| ***At* vs. *Vv*** |  | 0.149 | 0.739 |  | 0.385 | | 0.778 | |  | | 0.141 | | 0.953 | |
| ***At* vs. *Pt*** |  | 0.047 | 0.754 |  | 0.412 | | 0.770 | |  | | 0.020 | | 1.016 | |
| ***Mt* vs. *Vv*** |  | 0.053 | 0.659 |  | 0.373 | | 0.809 | |  | | 0.086 | | 0.841 | |
| ***Mt* vs. *Pt*** |  | 0.066 | 0.716 |  | 0.567 | | 0.834 | |  | | 0.044 | | 0.905 | |
|  | | | | | | | | | | | | | | |
| **Sub-dataset 2: PP2C gene family (24 Clades)** | | | | | | | | | | | | | | |
| **Comparison**  **Pair** |  | **d** | |  | **p_N_** | | | |  | | **p_S_** | | | |
|  |  | **R^2^** | **Slope** |  | **R^2^** | | **Slope** | |  | | **R^2^** | | **Slope** | |
| ***At* vs. *Vv*** |  | 0.637 | 0.810 |  | 0.804 | | 0.825 | |  | | 0.003 | | 0.922 | |
| ***At* vs. *Pt*** |  | 0.357 | 0.814 |  | 0.744 | | 0.767 | |  | | 0.000 | | 0.962 | |
| ***Mt* vs. *Vv*** |  | 0.387 | 0.775 |  | 0.539 | | 0.818 | |  | | 0.287 | | 0.915 | |
| ***Mt* vs. *Pt*** |  | 0.263 | 0.786 |  | 0.344 | | 0.742 | |  | | 0.261 | | 0.964 | |
|  |  |  |  |  | |  | |  | |  | |  | |  |
| **Sub-dataset 3: LRR-Pkinase gene family (52 Clades)** | | | | | | | | | | | | | | |
| **Comparison**  **Pair** |  | **d** | |  | **p_N_** | | | |  | | **p_S_** | | | |
|  |  | **R^2^** | **Slope** |  | **R^2^** | | **Slope** | |  | | **R^2^** | | **Slope** | |
| ***At* vs. *Vv*** |  | 0.471 | 0.775 |  | 0.669 | | 0.805 | |  | | 0.082 | | 0.941 | |
| ***At* vs. *Pt*** |  | 0.510 | 0.887 |  | 0.750 | | 0.872 | |  | | 0.000 | | 1.001 | |
| ***Mt* vs. *Vv*** |  | 0.091 | 0.673 |  | 0.677 | | 0.811 | |  | | 0.032 | | 0.864 | |
| ***Mt* vs. *Pt*** |  | 0.213 | 0.796 |  | 0.688 | | 0.881 | |  | | 0.134 | | 0.933 | |

**Table S7.** Sign-test for annual-perennial comparison of evolutionary rates, estimated by the ML method, for the 3 sub-datasets sampled from non-housekeeping gene families. The *P*-value of sign-test in all 4 annual-perennial cross-comparisons and the proportion of genes showing higher evolutionary rate in annuals than in perennials are listed in the table.

|  | | | | | | | | | | | | | | |
| --- | --- | --- | --- | --- | --- | --- | --- | --- | --- | --- | --- | --- | --- | --- |
| **Sub-dataset 1: 8 different gene families (35 Clades)** | | | | | | | | | | | | | | |
| **Comparison**  **Pair** |  | **d** | |  | **d_N_** | | | |  | | **d_S_** | | | |
|  |  | **Proportion** | **P-Value** |  | **Proportion** | | **P-Value** | |  | | **Proportion** | | **P-Value** | |
| ***At* vs. *Vv*** |  | 85.7% | 1.12E-05 |  | 65.7% | | 0.045 | |  | | 82.9% | | 5.84E-05 | |
| ***At* vs. *Pt*** |  | 71.4% | 8.34E-03 |  | 54.3% | | 0.368 | |  | | 77.1% | | 9.39E-04 | |
| ***Mt* vs. *Vv*** |  | 80.0% | 2.54E-04 |  | 77.1% | | 9.39E-04 | |  | | 68.6% | | 0.021 | |
| ***Mt* vs. *Pt*** |  | 65.7% | 0.045 |  | 71.4% | | 8.34E-03 | |  | | 60.0% | | 0.155 | |
|  | | | | | | | | | | | | | | |
| **Sub-dataset 2: PP2C gene family (24 Clades)** | | | | | | | | | | | | | | |
| **Comparison**  **Pair** |  | **d** | |  | **d_N_** | | | |  | | **d_S_** | | | |
|  |  | **Proportion** | **P-Value** |  | **Proportion** | | **P-Value** | |  | | **Proportion** | | **P-Value** | |
| ***At* vs. *Vv*** |  | 75.0% | 0.011 |  | 58.3% | | 0.271 | |  | | 83.3% | | 7.72E-04 | |
| ***At* vs. *Pt*** |  | 70.8% | 0.032 |  | 62.5% | | 0.154 | |  | | 83.3% | | 7.72E-04 | |
| ***Mt* vs. *Vv*** |  | 75.0% | 0.011 |  | 79.2% | | 3.31E-03 | |  | | 100.0% | | 0.000 | |
| ***Mt* vs. *Pt*** |  | 79.2% | 3.31E-03 |  | 66.7% | | 0.076 | |  | | 83.3% | | 7.72E-04 | |
|  |  |  |  |  | |  | |  | |  | |  | |  |
| **Sub-dataset 3: LRR-Pkinase gene family (52 Clades)** | | | | | | | | | | | | | | |
| **Comparison**  **Pair** |  | **d** | |  | **d_N_** | | | |  | | **d_S_** | | | |
|  |  | **Proportion** | **P-Value** |  | **Proportion** | | **P-Value** | |  | | **Proportion** | | **P-Value** | |
| ***At* vs. *Vv*** |  | 73.1% | 5.98E-04 |  | 55.8% | | 0.244 | |  | | 73.1% | | 5.98E-04 | |
| ***At* vs. *Pt*** |  | 76.9% | 6.38E-05 |  | 55.8% | | 0.244 | |  | | 78.8% | | 1.79E-05 | |
| ***Mt* vs. *Vv*** |  | 82.7% | 1.02E-06 |  | 82.7% | | 1.02E-06 | |  | | 80.8% | | 9.53E-06 | |
| ***Mt* vs. *Pt*** |  | 84.6% | 4.07E-07 |  | 73.1% | | 5.98E-04 | |  | | 80.8% | | 9.53E-06 | |

**Table S8.** Paired *t*-test for annual-perennial comparison of evolutionary rates, estimated by the ML method, in the 3 sub-datasets sampled from non-housekeeping gene families. The *P*-value of sign-test in all 4 annual-perennial cross-comparisons show higher evolutionary rate in annuals than in perennials.

|  | | | | | | | | | | | | | | |
| --- | --- | --- | --- | --- | --- | --- | --- | --- | --- | --- | --- | --- | --- | --- |
| **Sub-dataset 1: 8 different gene families (35 Clades)** | | | | | | | | | | | | | | |
| **Comparison**  **Pair** |  | **d** | |  | **d_N_** | | | |  | | **d_S_** | | | |
|  |  | ***Vv*** | ***Pt*** |  | ***Vv*** | | ***Pt*** | |  | | ***Vv*** | | ***Pt*** | |
| ***At*** |  | 4.18E-04 | 0.030 |  | 5.56E-03 | | 0.183 | |  | | 4.16E-03 | | 0.038 | |
| ***Mt*** |  | 7.18E-04 | 0.057 |  | 2.48E-03 | | 0.018 | |  | | 4.17E-03 | | 0.307 | |
|  | | | | | | | | | | | | | | |
| **Sub-dataset 2: PP2C gene family (24 Clades)** | | | | | | | | | | | | | | |
| **Comparison**  **Pair** |  | **d** | |  | **d_N_** | | | |  | | **d_S_** | | | |
|  |  | ***Vv*** | ***Pt*** |  | ***Vv*** | | ***Pt*** | |  | | ***Vv*** | | ***Pt*** | |
| ***At*** |  | 0.031 | 0.037 |  | 0.484 | | 0.480 | |  | | 0.038 | | 0.043 | |
| ***Mt*** |  | 6.39E-03 | 5.81E-04 |  | 0.075 | | 0.045 | |  | | 5.95E-03 | | 1.19E-03 | |
|  |  |  |  |  | |  | |  | |  | |  | |  |
| **Sub-dataset 3: LRR-Pkinase gene family (52 Clades)** | | | | | | | | | | | | | | |
| **Comparison**  **Pair** |  | **d** | |  | **d_N_** | | | |  | | **d_S_** | | | |
|  |  | ***Vv*** | ***Pt*** |  | ***Vv*** | | ***Pt*** | |  | | ***Vv*** | | ***Pt*** | |
| ***At*** |  | 1.61E-03 | 4.61E-04 |  | 0.011 | | 0.051 | |  | | 1.24E-03 | | 2.19E-04 | |
| ***Mt*** |  | 4.99E-06 | 1.12E-04 |  | 2.24E-07 | | 6.99E-05 | |  | | 2.07E-04 | | 1.04E-03 | |

**Table S9.** Correlation between annual-perennial evolutionary rates, estimated by the ML method, for the 3 sub-datasets sampled from non-housekeeping gene families based on the data estimated by the ML method. The square of correlation coefficient (R^2^) and the slope of regression line of evolutionary rate in annuals against that in perennials are listed in the table.

|  | | | | | | | | | | | | | | | |
| --- | --- | --- | --- | --- | --- | --- | --- | --- | --- | --- | --- | --- | --- | --- | --- |
| **Sub-dataset 1: 8 different gene families (35 Clades)** | | | | | | | | | | | | | | | |
| **Comparison**  **Pair** |  | **d** | |  | **d_N_** | | | |  | | **d_S_** | | | |  |
|  |  | **R^2^** | **Slope** |  | **R^2^** | | **Slope** | |  | | **R^2^** | | **Slope** | |  |
| ***At* vs. *Vv*** |  | 0.054 | 0.467 |  | 0.684 | | 0.814 | |  | | 0.000 | | 0.405 | |  |
| ***At* vs. *Pt*** |  | 0.025 | 0.588 |  | 0.658 | | 0.902 | |  | | 0.013 | | 0.520 | |  |
| ***Mt* vs. *Vv*** |  | 0.319 | 0.641 |  | 0.287 | | 0.552 | |  | | 0.071 | | 0.649 | |  |
| ***Mt* vs. *Pt*** |  | 0.475 | 0.847 |  | 0.301 | | 0.632 | |  | | 0.318 | | 0.941 | |  |
|  | | | | | | | | | | | | | | | |
| **Sub-dataset 2: PP2C gene family (24 Clades)** | | | | | | | | | | | | | | | |
| **Comparison**  **Pair** |  | **d** | |  | **d_N_** | | | |  | | **d_S_** | | | |  |
|  |  | **R^2^** | **Slope** |  | **R^2^** | | **Slope** | |  | | **R^2^** | | **Slope** | |  |
| ***At* vs. *Vv*** |  | 0.040 | 0.606 |  | 0.342 | | 0.959 | |  | | 0.000 | | 0.481 | |  |
| ***At* vs. *Pt*** |  | 0.026 | 0.604 |  | 0.453 | | 0.965 | |  | | 0.000 | | 0.482 | |  |
| ***Mt* vs. *Vv*** |  | 0.566 | 0.786 |  | 0.531 | | 0.840 | |  | | 0.549 | | 0.755 | |  |
| ***Mt* vs. *Pt*** |  | 0.781 | 0.829 |  | 0.650 | | 0.856 | |  | | 0.726 | | 0.798 | |  |
|  |  |  |  |  | |  | |  | |  | |  | |  | |
| **Sub-dataset 3: LRR-Pkinase gene family (52 Clades)** | | | | | | | | | | | | | | | |
| **Comparison**  **Pair** |  | **d** | |  | **d_N_** | | | |  | | **d_S_** | | | |  |
|  |  | **R^2^** | **Slope** |  | **R^2^** | | **Slope** | |  | | **R^2^** | | **Slope** | |  |
| ***At* vs. *Vv*** |  | 0.143 | 0.683 |  | 0.477 | | 0.830 | |  | | 0.070 | | 0.613 | |  |
| ***At* vs. *Pt*** |  | 0.394 | 0.738 |  | 0.540 | | 0.870 | |  | | 0.282 | | 0.666 | |  |
| ***Mt* vs. *Vv*** |  | 0.602 | 0.791 |  | 0.654 | | 0.767 | |  | | 0.517 | | 0.794 | |  |
| ***Mt* vs. *Pt*** |  | 0.605 | 0.834 |  | 0.546 | | 0.798 | |  | | 0.530 | | 0.829 | |  |

**Table S10.** Exception genes against the global trend of higher evolutionary rates in annuals than in perennials. The exception here is defined as showing consistent disagreements with the global trends of faster evolution tempo in annuals than in perennials in all 4 annual-perennial cross comparisons.

| **Gene catalog** | **Gene name** | **Estimators showing the exception** | | |
| --- | --- | --- | --- | --- |
|  |  | **d** | **d_N_** | **d_S_** |
| Nuclear housekeeping genes | Tranketolase | Yes | Yes | Yes |
|  | Prenylcysteine oxidase 1 | Yes | Yes | Yes |
|  | Glutaredoxin_2 | Yes | No | Yes |
|  | microsomal-glutathione-S-transferase 3 | No | Yes | No |
|  | Molybdopterin converting factor | No | Yes | No |
|  | Mitochondrial ribosomal protein L43 | No | Yes | No |
|  | NADP-dependent glyceraldehyde-3-phosphate dehydrogenase | No | Yes | No |
|  | protein_arginine_N-methyltransferase | No | Yes | No |
|  | ribulose-phosphate 3-epimerase | Yes | No | Yes |
|  |  |  |  |  |
| Chloroplast genes | psaC | Yes | Yes | Yes |

**Table S11.** Plant genome data used in this study.

| **Plant Species** | **DATA Version** | **Download Website** |
| --- | --- | --- |
| *Arabidopsis thaliana* | TAIR8 | http://www.arabidopsis.org/ |
| *Medicago truncatula* | Mt2.0 | <http://www.medicago.org/> |
| *Vitis vinifera* | v1.0 | http://www.genoscope.cns.fr/externe/Download/Projets/Projet_ML/data/ |
| *Populus trichocarpa* | v1.1 | http://genome.jgi-psf.org/Poptr1_1/Poptr1_1.home.html |
| *Oryza sativa* | v6.0 | http://rice.plantbiology.msu.edu/ |

**Table S12.** Housekeeping genes sampled in this study

| **Housekeeping Gene Loci**  **(85 in total)** | **Outgroup** | **Annuals** | | **Perennials** | |
| --- | --- | --- | --- | --- | --- |
|  | ***O.sativa*** | ***A.thalaina*** | ***M.truncatula*** | ***V.vinifera*** | ***P.trichocarpa*** |
| 15 kDa selenoprotein | Os01g66960 | AT1G05720 | AC165439_17.2 | GSVIVT00034661001 | 811353;665871 |
| 16S rRNA-processing protein | Os05g49130 | AT5G46420 | AC144608_51.2 | GSVIVT00003385001 | 278191 |
| 2,3-biphosphoglycerate-independent phosphoglycerate mutase | Os05g40420 | AT1G09780;AT3G08590 | AC124215_22.2 | GSVIVT00033622001 | 739764;825441 |
| 39S ribosomal protein L46 | Os06g19640 | AT1G14620 | AC152407_30.2 | GSVIVT00026907001 | 644529 |
| 3-deoxy-D-manno-octulosonic-acid transferase | Os01g63840 | AT5G03770 | AC146788_6.2 | GSVIVT00021705001 | 576764 |
| 3-phosphoshikimate 1-carboxyvinyltransferase | Os06g04280 | AT1G48860;AT2G45300 | AC127674_14.2;AC119419_4.2 | GSVIVT00026409001 | 244003, 552035 |
| 50S ribosomal protein L4 | Os06g03790 | AT1G07830 | CR962127_26.2 | GSVIVT00016145001 | 731548;644722 |
| 5'-nucleotidase, cytosolic III | Os03g44660 | AT2G38680 | AC175312_41.2;AC157982_11.2 | GSVIVT00002651001 | 567878;280391 |
| alkaline phytoceramidase | Os03g49180 | AT4G22330 | AC148915_1.2 | GSVIVT00005251001;GSVIVT00038830001 | 837466;286147;568183;752844;597054 |
| arp2_3 complex 20 kd subunit | Os03g63090 | AT4G14147 | AC130798 32.2 | GSVIVT00038837001 | 827538 |
| aspartyl_glutamyl-tRNA amidotransferase subunit B | Os11g34210 | AT1G48520 | CR962123_5.2 | GSVIVT00020369001 | 766505;833424 |
| ataxin 10 | Os03g02720 | AT4G00231 | AC169173_10.2 | GSVIVT00034822001 | 572162;564893 |
| ATP binding protein kinase | Os02g46750 | AT5G16810 | AC145222_58.2 | GSVIVT00014006001 | 738378 |
| ATPase, H+ transporting, lysosomal 42kDa, V1 subunit C1 | Os05g51530 | AT1G12840 | AC149131_49.2 | GSVIVT00018019001;GSVIVT00037933001 | 174695;810676; |
| brain protein 16 | Os10g26660 | AT1G14300 | AC146568_4.2 | GSVIVT00036985001 | 574363, 745075 |
| carboxypeptidase D | Os06g05240 | AT1G71696 | AC148397_36.2 | GSVIVT00008960001 | 761088 |
| centromerekinetochore protein zw10 | Os11g34310 | AT2G32900 | CT573055_41.2 | GSVIVT00025970001 | 552728 |
| Citrate synthase_glyoxysomal | Os02g13840 | AT2G42790;AT3G58740;AT3G58750 | CT009528_19.2 | GSVIVT00018944001 | 732045;735490 |
| CRR6 (CHLORORESPIRATORY REDUCTION 6) | Os08g07060 | AT2G47910 | AC138131_41.2 | GSVIVT00005655001 | 272637;578826 |
| dephospho-CoA kinase | Os01g25880 | AT2G27490 | AC146855_19.2 | GSVIVT00027960001 | 805521;430704 |
| DiGeorge syndrome critical region protein 14 | Os06g30710 | AT3G07790 | CR955006_19.2 | GSVIVT00029145001 | 552692;775129 |
| dihydroxy-acid dehydratase | Os08g44530 | AT3G23940 | AC165430_29.2 | GSVIVT00020486001 | 830949;814873 |
| DNA binding / hydrolase, acting on ester bonds / nuclease/ recombinase | Os10g40650 | AT1G12244 | AC174366_2.2;AC174366_27.2 | GSVIVT00019198001 | 783184 |
| DNA damage checkpoint protein | Os04g44620 | AT1G52530 | AC136503_51.2 | GSVIVT00000292001 | 597617 |
| DNA primase large subunit | Os07g22400 | AT1G67320 | AC144644_34.2 | GSVIVT00020497001 | 712440 |
| DNA replication complex GINS protein PSF1 | Os05g37980 | AT1G80190 | AC125481_21.2 | GSVIVT00034589001 | 589281 |
| electron carrier iron ion binding | Os09g33950 | AT3G07480 | AC171166_6.2 | GSVIVT00013308001 | 553127;824407 |
| EMB1381 (EMBRYO DEFECTIVE 1381) | Os09g12500 | AT2G31340 | CU104689_3.1 | GSVIVT00025950001 | 755854 |
| EMB2752 (EMBRYO DEFECTIVE 2752) | Os05g46280 | AT4G29660 | AC195571_3.1;AC146632_4.2 | GSVIVT00016577001 | 661486 |
| exosomal core protein CSL4 | Os02g57070 | AT5G38890 | AC173834_6.2 | GSVIVT00026698001 | 663010 |
| Fumarase | Os03g21950 | AT2G47510;AT5G50950 | AC152751_10.2 | GSVIVT00022670001;GSVIVT00028048001 | 552480 |
| galactose-1-phosphate uridylyltransferase | Os07g07550 | AT5G18200 | AC174375_4.2; AC174375_25.2 | GSVIVT00025477001 | 561702;826137 |
| general transcription factor IIH_polypeptide 2_44kDa-like | Os04g42990 | AT1G05055 | AC135795_1.2 | GSVIVT00024501001 | 837441 |
| glucose-6-phosphate isomerase, cytosolic (PGIC) | Os03g56460 | AT5G42740 | AC137080_15.2 | GSVIVT00014624001 | 431847 |
| Glutaredoxin 2 | Os02g52900 | AT4G08280 | CR538723_8.2 | GSVIVT00015366001 | 410048;581167 |
| glutathione-dependent formaldehyde-activating, GFA | Os09g23370 | AT5G16940 | AC148818_14.2 | GSVIVT00031191001 | 645201;552923;645200 |
| glycosylphosphatidylinositol anchor attachment 1 (GPAA1) | Os01g48980 | AT5G19130 | AC152349_8.2 | GSVIVT00020667001 | 173974 |
| GTP cyclohydrolase I | Os04g56710 | AT3G07270 | AC171804_14.2; | GSVIVT00036941001;GSVIVT00005777001 | 573397;553210 |
| guanylyl cyclase | Os05g37950 | AT5G05930 | CT963133_7.2 | GSVIVT00018367001 | 720589 |
| HAD hydrolase-like protein/gas vesicle protein K | Os07g23520 | AT2G45990 | AC139601_31.2;AC139601_17.2 | GSVIVT00026887001 | 731114 |
| histidinol dehydrogenase | Os01g13190 | AT5G63890 | AC192958_17.1 | GSVIVT00002274001 | 837236;550607 |
| Indole 3-glycerolphosphate synthase | Os04g39270 | AT5G48220;AT2G04400 | CR938710_11.2 | GSVIVT00029073001 | 834976 |
| LEAFY protein | Os04g51009 | AT5G61850 | AC139708_6.2 | GSVIVT00018048001 | 835248 |
| MAK10 homolog, amino-acid N-acetyltransferase subunit | Os02g55950 | AT2G11000 | AC182813_30.2 | GSVIVT00016969001 | 560565 |
| MGC83919 protein | Os02g01710 | AT5G24680 | CR932039_38.2 | GSVIVT00037130001 | 772586 |
| microsomal glutathione S-transferase 3 | Os03g50130 | AT1G65820 | CR955011_29.2 | GSVIVT00023521001 | 831173;289216 |
| mitochondrial ribosomal protein L43 | Os03g10930 | AT3G59650 | AC151460_6.2 | GSVIVT00034657001 | 814669 |
| molybdenum cofactor biosynthesis protein C | Os04g38700 | AT1G01290 | AC146856 5.2 | GSVIVT00008624001 | 572402 |
| molybdopterin biosynthesis MoeA protein | Os04g56620 | AT5G20990 | CR954186_7.2 | GSVIVT00017434001 | 556880 |
| molybdopterin converting factor, subunit 2 | Os02g04740 | AT2G43760 | AC147363_12.2 | GSVIVT00017312001;GSVIVT00017321001 | 706487 |
| NADP+ Isocitrate dehydrogenase | Os01g46610;Os05g49760;Os01g14580 | AT1G54340;AT1G65930 | AC132565_27.2;AC126787_7.2 | GSVIVT00002132001;GSVIVT00023479001 | 803363;770098;678577;648243 |
| NADP-dependent glyceraldehyde-3-phosphate dehydrogenase | Os08g34210 | AT2G24270 | AC169120_18.2;AC160629_12.2 | GSVIVT00032695001;GSVIVT00017234001 | 261294;578767;717030 |
| NCK-associated protein 1 | Os08g43130 | AT2G35110 | AC147481_50.2 | GSVIVT00030153001 | 575639;575636;662489 |
| NTA15 protein | Os05g05600 | AT1G66330 | AC155282_17.2 | GSVIVT00022467001 | 816844 |
| nucleoporin 85 | Os01g54240 | AT4G32910 | AC202471_14.1 | GSVIVT00032420001 | 825675 |
| peptidyl-prolyl cis-transisomerase, cyclophilin type | Os03g01090 | AT4G17070 | AC146572_12.2;AC149129_19.2 | GSVIVT00010718001;GSVIVT00019047001 | 580156;589929;781338;420821 |
| phenylalanyl-tRNA synthetase, beta subunit | Os05g48510 | AT1G72550 | AC202304_14.1 | GSVIVT00017573001 | 828230;730088 |
| phosphatidyl synthase | Os03g17590 | AT3G45740 | AC137829_20.2 | GSVIVT00018280001;GSVIVT00024446001 | 765354;567538 |
| Phosphoribosylanthranilate isomerase | Os02g16630 | AT1G07780;AT5G05590;AT1G29410 | CT573029_27.2 | GSVIVT00029306001 | 564037;728534 |
| phosphotyrosyl phosphatase activator | Os06g11640 | AT4G08960 | AC144515_30.2;AC202334_13.1 | GSVIVT00015198001;GSVIVT00015211001 | 760756;551596;651263 |
| photosystem 1 subunit 5 | Os09g30340 | AT1G55670 | CU207236_16.1 | GSVIVT00003325001 | 550669;744851 |
| phytoene synthase | Os06g01470 | AT1G62730 | CT573076_29.2 | GSVIVT00028617001 | 837085 |
| prenylcysteine oxidase 1 | Os04g59630 | AT5G63910 | CR936364_68.2 | GSVIVT00019298001 | 764062;789008 |
| proprotein convertase | Os09g25650 | AT5G49150 | CT033768_30.2 | GSVIVT00033029001 | 790135;763865 |
| proteasome (prosome, macropain) 26S subunit, ATPase 3, interacting protein | Os03g50220 | AT1G13330 | CU302348_13.1 | GSVIVT00030318001 | 564514;769669 |
| protein arginine N-methyltransferase | Os02g04660 | AT4G31120 | AC140026_8.2 | GSVIVT00032396001 | 835729;271696;290529 |
| ribosomal protein L28 | Os07g36090 | AT4G31460 | AC202483_7.1 | GSVIVT00014400001 | 261674 |
| ribosome-binding factor A | Os06g04610 | AT4G34730 | CU024880_45.2 | GSVIVT00035981001 | 800817 |
| ribulose-phosphate 3-epimerase (IC) | Os03g13450 | AT1G66080 | AC149209_15.2 | GSVIVT00026683001 | 831166 |
| S-adenosylmethionine synthetase | Os05g04510 | AT3G17390;AT4G01850;AT1G02500 | AC144563_19.2;AC174352_23.2;AC202321_13.1 | GSVIVT00028192001;GSVIVT00022531001;GSVIVT00019707001 | 564333;724015;729359;834837;644907 |
| SNO glutamine amidotransferase | Os02g03740 | AT5G60540 | AC138016_17.2 | GSVIVT00024517001 | 723560 |
| Succinyl-CoA synthetase a-chain | Os07g38970 | AT5G08300;AT5G23250 | AC157350_25.2 | GSVIVT00031891001;GSVIVT00037069001 | 831388;827745;433489 |
| tetraacyldisaccharide 4'-kinase | Os07g01200 | AT3G20480 | CT962504_12.2 | GSVIVT00027306001 | 242629 |
| thylakoid lumen 18.3 kDa protein | Os05g33280 | AT1G54780 | AC174299_17.2 | GSVIVT00022877001 | 570885;836765 |
| Thylakoid lumenal 17.9 kDa protein, chloroplast precursor | Os01g01280 | AT4G24930 | CT010521_25.2 | GSVIVT00017961001 | 575370 |
| thymidylate kinase | Os01g70950 | AT4G03200 | AC198007_25.1 | GSVIVT00023575001 | 824472 |
| thyroid hormone receptor interactor 4 | Os03g64250 | AT2G20410 | AC146861_41.2 | GSVIVT00002198001;GSVIVT00020251001;GSVIVT00020249001 | 248095;571088 |
| TOM2B (tobamovirus multiplication protein 2B) | Os02g17850 | AT1G32370 | AC144430_21.2 | GSVIVT00015831001 | 589976 |
| Tranketolase | Os06g04270;  Os04g19740 | AT3G60750  AT2G45290 | AC146329_8.2;AC119419_30.2 | GSVIVT00038435001; GSVIVT00026404001 | 552034;730849 |
| Transaldolase | Os08g05830 | AT1G12230 | AC155228_11.2 | GSVIVT00007295001 | 267155 |
| transcriptional activator, TenA family | Os03g19390 | AT3G16990 | AC126780_53.2 | GSVIVT00033127001 | 656656 |
| Transmembrane protein 103 | Os03g17560 | AT4G10090 | AC196765_2.1 | GSVIVT00029487001 | 832517 |
| tRNA a64-2'-o-ribosylphosphate transferase | Os06g36170 | AT2G40570 | AC152919_28.2 | GSVIVT0001666600 | 809250 |
| Vcell division cycle 73, Paf1 RNA polymerase II complex component | Os03g46190 | AT3G22590 | CU302334_26.1 | GSVIVT00020021001 | 769296;593408 |
| xylose isomerase | Os07g47290 | AT5G57655 | AC127169_49.2 | GSVIVT00031456001 | 737826 |

**Table S13.** Chloroplast genes sampled in this study.

| Gene loci  (34 in total) | Outgroup | Annuals | | Prennials | |
| --- | --- | --- | --- | --- | --- |
|  | *O. sativa* | *A. thalaina* | *M. truncatula* | *V. vinifera* | *P. trichocarpa* |
| atpA | Rice atpA | Arabidopsis atpA | Medicago atpA | Grape atpA | Poplar atpA |
| atpB | Rice atpB | Arabidopsis atpB | Medicago atpB | Grape atpB | Poplar atpB |
| atpF | Rice atpF | Arabidopsis atpF | Medicago atpF | Grape atpF | Poplar atpF |
| atpI | Rice atpI | Arabidopsis atpI | Medicago atpI | Grape atpI | Poplar atpI |
| ccsA | Rice ccsA | Arabidopsis ccsA | Medicago ccsA | Grape ccsA | Poplar ccsA |
| cemA | Rice cemA | Arabidopsis cemA | Medicago cemA | Grape cemA | Poplar cemA |
| clpP | Rice clpP | Arabidopsis clpP | Medicago clpP | Grape clpP | Poplar clpP |
| matK | Rice matK | Arabidopsis matK | Medicago matK | Grape matK | Poplar matK |
| ndhD | Rice ndhD | Arabidopsis ndhD | Medicago ndhD | Grape ndhD | Poplar ndhD |
| ndhF | Rice ndhF | Arabidopsis ndhF | Medicago ndhF | Grape ndhF | Poplar ndhF |
| ndhG | Rice ndhG | Arabidopsis ndhG | Medicago ndhG | Grape ndhG | Poplar ndhG |
| petA | Rice petA | Arabidopsis petA | Medicago petA | Grape petA | Poplar petA |
| petB | Rice petB | Arabidopsis petB | Medicago petB | Grape petB | Poplar petB |
| psaA | Rice psaA | Arabidopsis psaA | Medicago psaA | Grape psaA | Poplar psaA |
| psaB | Rice psaB | Arabidopsis rpoB | Medicago rpoB | Grape rpoB | Poplar rpoB |
| psaC | Rice psaC | Arabidopsis rpoC1 | Medicago rpoC1 | Grape rpoC1 | Poplar rpoC1 |
| psbB | Rice psbB | Arabidopsis psbB | Medicago psbB | Grape psbB | Poplar psbB |
| psbC | Rice psbC | Arabidopsis psbC | Medicago psbC | Grape psbC | Poplar psbC |
| psbN | Rice psbN | Arabidopsis psbN | Medicago psbN | Grape psbN | Poplar psbN |
| rbcL | Rice rbcL | Arabidopsis rbcL | Medicago rbcL | Grape rbcL | Poplar rbcL |
| rpl2 | Rice rpl2 | Arabidopsis rpl2 | Medicago rpl2 | Grape rpl2 | Poplar rpl2 |
| rpl14 | Rice rpl14 | Arabidopsis rpl14 | Medicago rpl14 | Grape rpl14 | Poplar rpl14 |
| rpl16 | Rice rpl16 | Arabidopsis rpl16 | Medicago rpl16 | Grape rpl16 | Poplar rpl16 |
| rpl23 | Rice rpl23 | Arabidopsis rpl23 | Medicago rpl23 | Grape rpl23 | Poplar rpl23 |
| rpl33 | Rice rpl33 | Arabidopsis rpl33 | Medicago rpl33 | Grape rpl33 | Poplar rpl33 |
| rpoA | Rice rpoA | Arabidopsis rpoA | Medicago rpoA | Grape rpoA | Poplar rpoA |
| rpoB | Rice rpoB | Arabidopsis rpoB | Medicago rpoB | Grape rpoB | Poplar rpoB |
| rpoC1 | Rice rpoC1 | Arabidopsis rpoC1 | Medicago rpoC1 | Grape rpoC1 | Poplar rpoC1 |
| rpoC2 | Rice rpoC2 | Arabidopsis rpoC2 | Medicago rpoC2 | Grape rpoC2 | Poplar rpoC2 |
| rps3 | Rice rps3 | Arabidopsis rps3 | Medicago rps3 | Grape rps3 | Poplar rps3 |
| rps8 | Rice rps8 | Arabidopsis rps8 | Medicago rps8 | Grape rps8 | Poplar rps8 |
| rps11 | Rice rps11 | Arabidopsis rps11 | Medicago rps11 | Grape rps11 | Poplar rps11 |
| rps14 | Rice rps14 | Arabidopsis rps14 | Medicago rps14 | Grape rps14 | Poplar rps14 |
| rps19 | Rice rps19 | Arabidopsis rps19 | Medicago rps19 | Grape rps19 | Poplar rps19 |

**Table S14.** Non-housekeeping gene families sampled in this study.

| **Gene Family Name** | **Pfam ID** | **Numbers of homolog genes in our sampled 5 plant species** | | | | |
| --- | --- | --- | --- | --- | --- | --- |
|  |  | **A.thaliana** | **M.truncatula** | **V.vinifera** | **P.trichocarpa** | **O.sativa** |
| 14-3-3 family | PF00244 | 14 | 7 | 9 | 12 | 8 |
| ARF transcription factor family | PF06507 | 22 | 13 | 20 | 37 | 25 |
| AS2 family | PF03195 | 42 | 24 | 45 | 57 | 36 |
| EXO70 exocyst subunit family | PF03081 | 23 | 17 | 15 | 29 | 42 |
| FH2 protein (formin) gene family | PF02181 | 20 | 11 | 13 | 22 | 17 |
| PP2C-type phosphatase gene family | PF00481 | 75 | 50 | 62 | 105 | 83 |
| ZF-HD transcription factor family | PF04770 | 11 | 3 | 5 | 13 | 6 |
| Xyloglucan Fucosyltransferase1family | PF03254 | 9 | 2 | 2 | 7 | 18 |
| LRR-Pkinase gene Family | PF00069 | 218 | 120 | 277 | 324 | 312 |
| AUX/IAA family | PF02309 | 29 | 8 | 24 | 33 | 29 |
