# Supplementary material for: Genome-wide investigation reveals high evolutionary rates in annual model plants
Source: BMC Plant Biol. 2010 Nov 9;10:242. doi: 10.1186/1471-2229-10-242 (PMC3095324; doi:10.1186/1471-2229-10-242)
Supplement: Additional file 2 — Supplemental Figures. Including all supplemental figures. Figure S1. Scatter plots of evolutionary rate of annuals against that of perennials for both nuclear and chloroplast genes estimated by the outgroup-dependent method. Cases in all 4 annual-perennial cross-comparison are shown. The dash line is the diagonal line with a slope equals to 1, and the red line is the regression line. Figure S2. Scatter plots of evolutionary rates of annuals against that of perennials for all 3 sub-datasets of non-housekeeping gene families estimated by the outgroup-dependent method. Cases in all 4 annual-perennial cross-comparison are shown. The dash line is the diagonal line with a slope equals to 1, and the red line is the regression line Figure S3. Scatter plots of evolutionary rate in annuals against that in perennials for the 3 sub-datasets collected from non-housekeeping gene families estimated by the ML method. Cases in all 4 annual-perennial cross-comparisons are shown. The dash line is the diagonal line with a slope equals to 1, and the red line is the regression line [file 1471-2229-10-242-S2.PPT]

## Slide 1
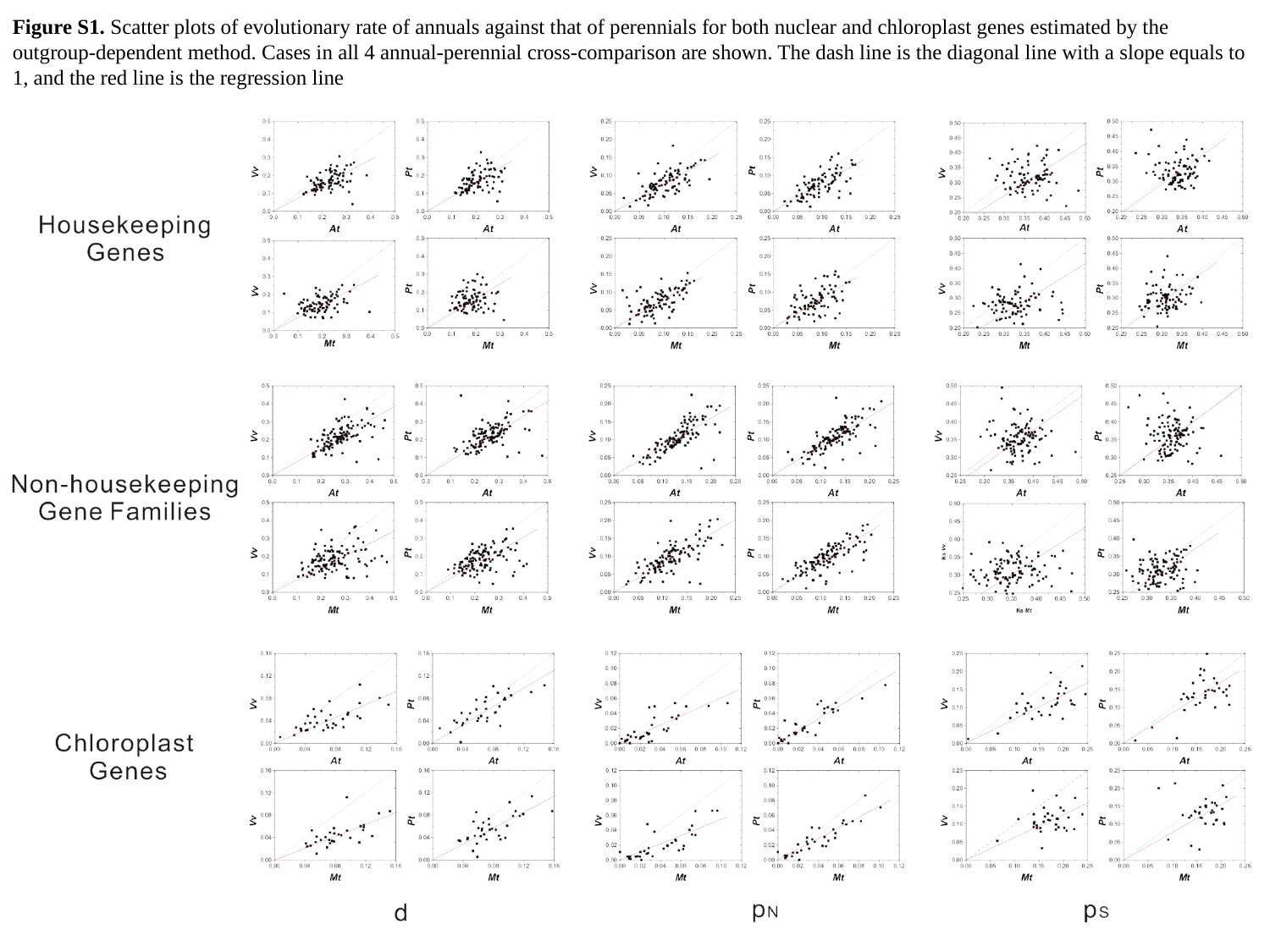

Figure S1. Scatter plots of evolutionary rate of annuals against that of perennials for both nuclear and chloroplast genes estimated by the outgroup-dependent method. Cases in all 4 annual-perennial cross-comparison are shown. The dash line is the diagonal line with a slope equals to 1, and the red line is the regression line

## Slide 2
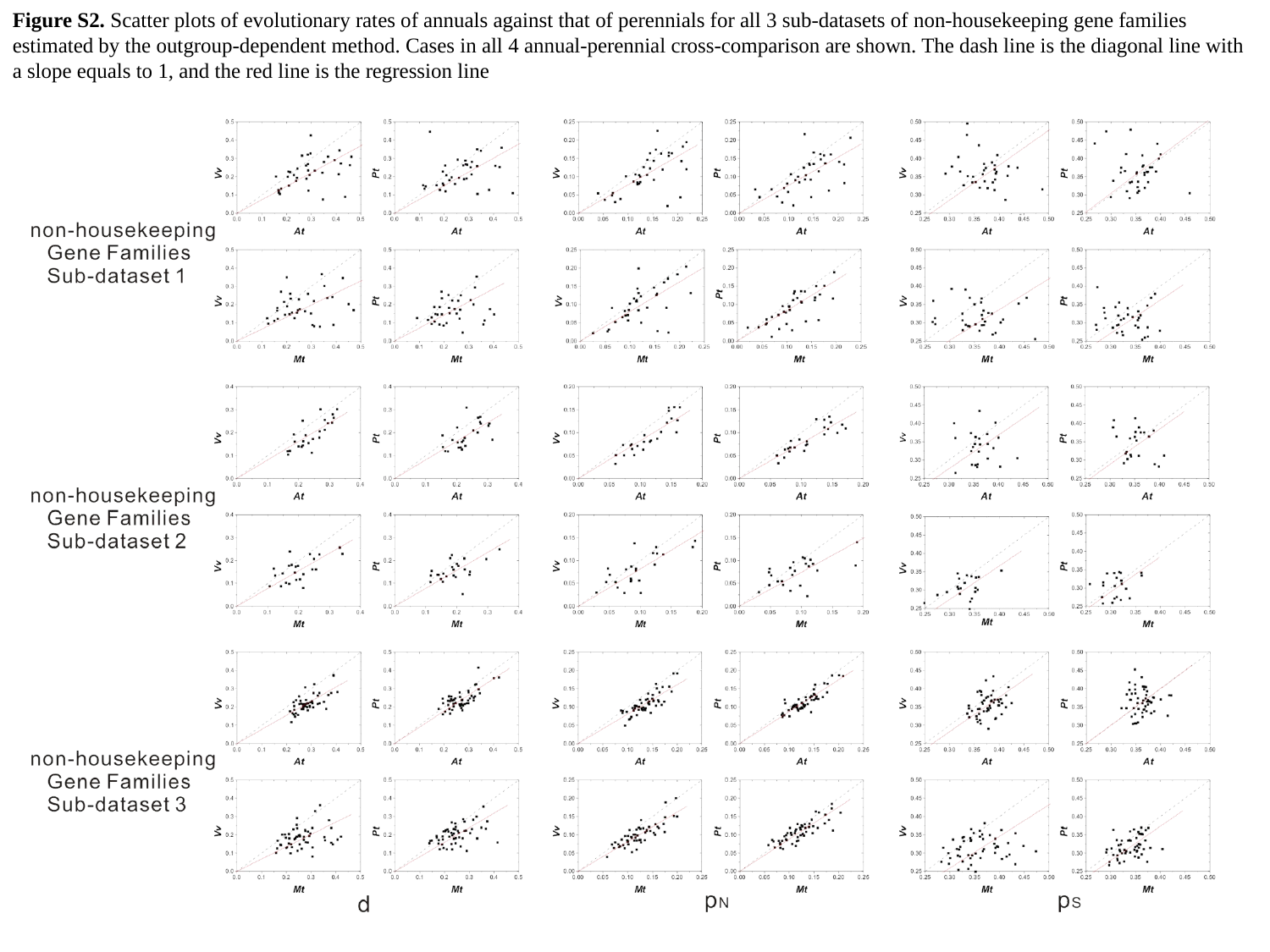

Figure S2. Scatter plots of evolutionary rates of annuals against that of perennials for all 3 sub-datasets of non-housekeeping gene families estimated by the outgroup-dependent method. Cases in all 4 annual-perennial cross-comparison are shown. The dash line is the diagonal line with a slope equals to 1, and the red line is the regression line

## Slide 3
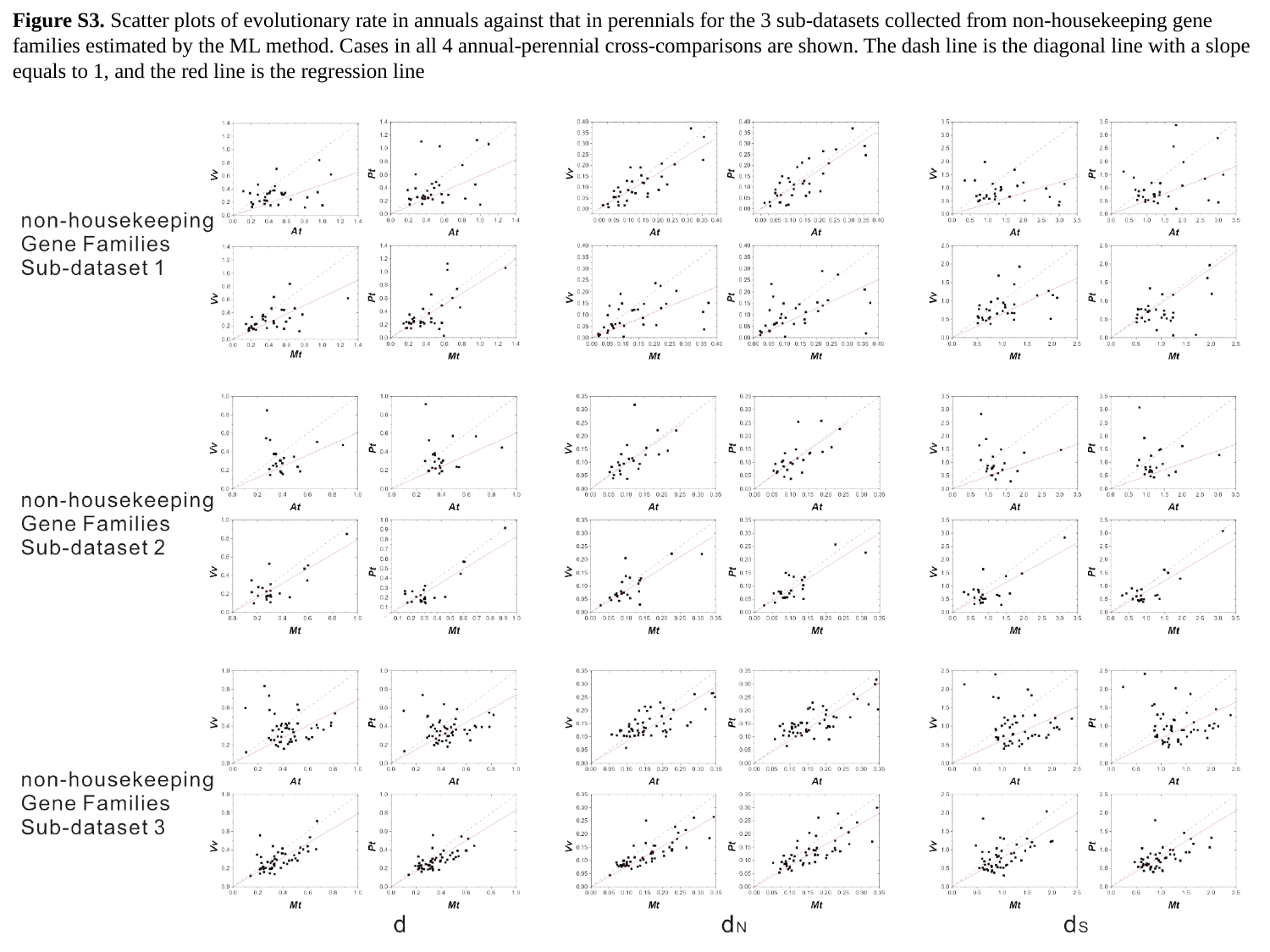

Figure S3. Scatter plots of evolutionary rate in annuals against that in perennials for the 3 sub-datasets collected from non-housekeeping gene families estimated by the ML method. Cases in all 4 annual-perennial cross-comparisons are shown. The dash line is the diagonal line with a slope equals to 1, and the red line is the regression line
